# Identifying the plasma metabolome responsible for mediating immune cell action in severe COVID-19: a Mendelian randomization investigation

**DOI:** 10.3389/fcimb.2024.1393432

**Published:** 2024-08-19

**Authors:** Yixia Zhang, Jie Hua, Liang Chen

**Affiliations:** ^1^ Department of Hematology, Nanjing Lishui People’s Hospital, Zhongda Hospital Lishui Branch, Southeast University, Nanjing, China; ^2^ Department of Gastroenterology, Jiangsu Province People’s Hospital, Nanjing, China; ^3^ Department of Infectious Diseases, Taikang Xianlin Drum Tower Hospital, Affiliated Hospital of Medical College of Nanjing University, Nanjing, China

**Keywords:** immune cells, plasma metabolome, severe COVID-19, mediator, Mendelian randomization

## Abstract

**Introduction:**

The immune response regulates the severity of COVID-19 (sCOVID-19). This study examined the cause-and-effect relationship between immune cell traits (ICTs) and the risk of severe COVID-19. Additionally, we discovered the potential role of plasma metabolome in modulating this risk.

**Methods:**

Employing data from a genome-wide association study (GWAS), we conducted a two-sample Mendelian randomization (MR) assessment of 731 genetic ICTs and sCOVID-19 (5,101 cases, 1,383,241 controls) incidence. The MR analysis was utilized to further quantitate the degree of plasma metabolome-mediated regulation of immune traits in sCOVID-19.

**Results:**

The inverse variance weighted method recognized 2 plasma metabolites (PMs) responsible for casual associations between immune cells and sCOVID-19 risk. These included Tridecenedioate (C13:1-DC) which regulated the association between CD27 on IgD- CD38br (OR 0.804, 95% CI 0.699–0.925, p = 0.002) and sCOVID-19 risk (mediated proportion: 18.7%); arginine to citrulline ratio which controlled the relationship of CD39 on monocyte (OR 1.053, 95% CI 1.013–1.094, p = 0.009) with sCOVID-19 risk (mediated proportion: -7.11%). No strong evidence that genetically predicted sCOVID-19 influenced the aforementioned immune traits.

**Conclusion:**

In this study, we have successfully identified a cause-and-effect relationship between certain ICTs, PMs, and the likelihood of contracting severe COVID-19. Our findings can potentially improve the accuracy of COVID-19 prognostic evaluation and provide valuable insights into the underlying mechanisms of the disease.

## Introduction

Since 2019, Corona Virus Disease 2019 (COVID-19) has seriously impacted the global population through its rapid transmission and lethal outcome (Initiative, 2020). As of January 2024, the World Health Organization (WHO) reported a total of 774,469,939 confirmed cases of COVID-19 and 7,026,465 deaths (Ablasser et al., 2013). Most patients displayed mild to severe symptoms, including fever, cough, and dyspnea. In addition, around 5–20% of patients experienced severe or critical conditions characterized by acute respiratory distress syndrome, severe hypoxemia, and acute lung damage, leading to fatal consequences (Initiative, 2020). Among severe COVID-19 (sCOVID-19) patients experiencing respiratory failure necessitating ventilation (i.e., arterial oxygen pressure to a fraction of the inspired oxygen ratio ≦ 200 mmHg), the mortality rate reached 30–60% (Initiative, 2020; Ihongbe et al., 2024). Although the condition has severe consequences, its signalling networks have not yet been identified.

Immune response critically modulates the pathogenesis of COVID-19 (Merad et al., 2022). Appropriate responses of the human innate and adaptive immunity against viruses, such as the induction of multiple immune cells and inflammatory cytokine subsets, are critical for combating viral replication and spread, restricting inflammation and eliminating infected cells (Torres-Ruiz et al., 2021). Dysregulated immunity or inflammatory responses typically cause extensive tissue damage. Metabolomic profiling can also impact disease risk and potentially serve as therapy targets. Prior investigations revealed a COVID-19-metabolome based on clinicopathological manifestations, immune status, and disease severity. COVID-19 severity is reported to be linked to dysregulated metabolic networks that are directly or indirectly correlated with the immune and systemic inflammatory response evident in COVID-19 patients (Orru et al., 2020).

Furthermore, plasma metabolites (PMs), namely, tryptophan, kynurenine and 3-hydroxykynurenine (i.e. PMs belonging to the kynurenine axis), can precisely estimate the COVID-19 disease course. A recent investigation by Suguru Saito et al. showed a significant reduction in tryptophan but elevation of kynurenine in ICU-admitted COVID-19 patients. Kynurenine promotes PD-L1 expression in B cells, correlating with increased IL-6R expression and STAT1/STAT3 activation (Saito et al., 2024a). Persistent metabolomic abnormalities were also observed in long and acute COVID patients (Saito et al., 2024b).

Mendelian randomization (MR) is a robust causal extrapolation tool that employs genetic variation (GV) as an instrumental variable (IV) to elucidate the exposure factor-mediated regulation of patient outcomes in observational studies (Li et al., 2023). Due to the arbitrary allocation of alleles during conception, this randomization procedure effectively accounts for confounding circumstances and decreases the likelihood of confounding. In this study, we conducted a two-sample Mendelian randomization (2S-MR) analysis to investigate the following: (i) Assessing the relationship between ICTs and the risk of sCOVID-19. (ii) Determining the importance of specific plasma metabolome profiles in influencing the effects.

## Materials and methods

### Research design

In this study, we investigated the reciprocal causal relationship between ICTs and the risk of sCOVID-19 using bidirectional 2S-MR. In this study, single nucleotide polymorphisms (SNPs) were called IVs. MR employs GV to denote risk factors. Hence, potential IVs must satisfy the following three assumptions: (1) GV is intricately linked to exposure; (2) GV has no relation with potential confounders between exposure and outcome; and (3) GV does not impact patient outcome using networks that do not involve exposure. Lastly, considering the lack of a consensus on sCOVID-19 definition, we identified sCOVID-19 as requiring invasive and noninvasive ventilation (Initiative, 2020).

### GWAS summary data sources

The employed ICT data were collected from the open-access GWAS database, containing information from a purely European population (GCST0001391 to GCST0002121) (Orru et al., 2020). In all, 731 ICTs were examined; namely, absolute cell (AC) counts (n = 118), median fluorescence intensities (MFI) indicating surface antigen levels (n = 389), morphological parameters [MP] (n = 32), and relative cell (RC) counts (n = 192). In particular, the MFI, AC and RC profiles included B cells, CDCs, mature T cells, monocytes, myeloid cells, TBNK (T cells, B cells, natural killer cells), and Treg panels. In contrast, the MP profile included CDC and TBNK panels. The original GWAS examined 3,757 European individuals to collect immunologic trait data, with no overlapping cohorts. To evaluate correlations after covariate adjustment (i.e., sex, age, and age), approximately 22 million SNPs genotyped using high-density arrays were incorporated into a Sardinian sequence-based reference panel. sCOVID-19 (GWAS ID: ebi-a-GCST011075) data were plotted from IEU OpenGWAS (https://gwas.mrcieu.ac.uk/datasets/ebi-a-GCST011075/), which contained 5101 sCOVID-19 cases and 1,383,241 controls. The plasma metabolome GWAS dataset contained 1,091 metabolites and 309 metabolite ratios among 8,299 individuals obtained from the Canadian Longitudinal Study on Aging (CLSA) cohort (Chen et al., 2023). All GWAS information was collected from varying consortia and organizations, providing no sample overlap.

### IV selection and data harmonization

The analysis included SNPs with genome-wide significance (P < 5 × 10^−8^). Without marked genome-wide SNPs as IVs, SNPs with P < 5 × 10^-6^ served as candidate IVs. Subsequently, SNPs underwent grouping according to the linkage disequilibrium (window size = 10,000 kb and r^2^ < 0.001). Linkage disequilibrium estimation was made according to the 1000 Genomes Project based on a European sample (Genomes Project et al., 2010). If a particular exposed SNP was not included in the outcome dataset, alternative SNPs were tagged using LD. Palindromic and ambiguous SNPs were omitted from the MR analysis (Genomes Project et al., 2010). The F statistic was computed via SNP-explained variance for individual exposures, i.e.[(N-K-1)/K]/[R^2^/(1- R^2^)], where R^2^ represents the proportion of variance in the exposure explained by the genetic variants, K represents the genetic variant quantity, and N represents the sample size. We eliminated weak IVs (F-statistics < 10).

### MR and mediation analyses

**Figure 1:**
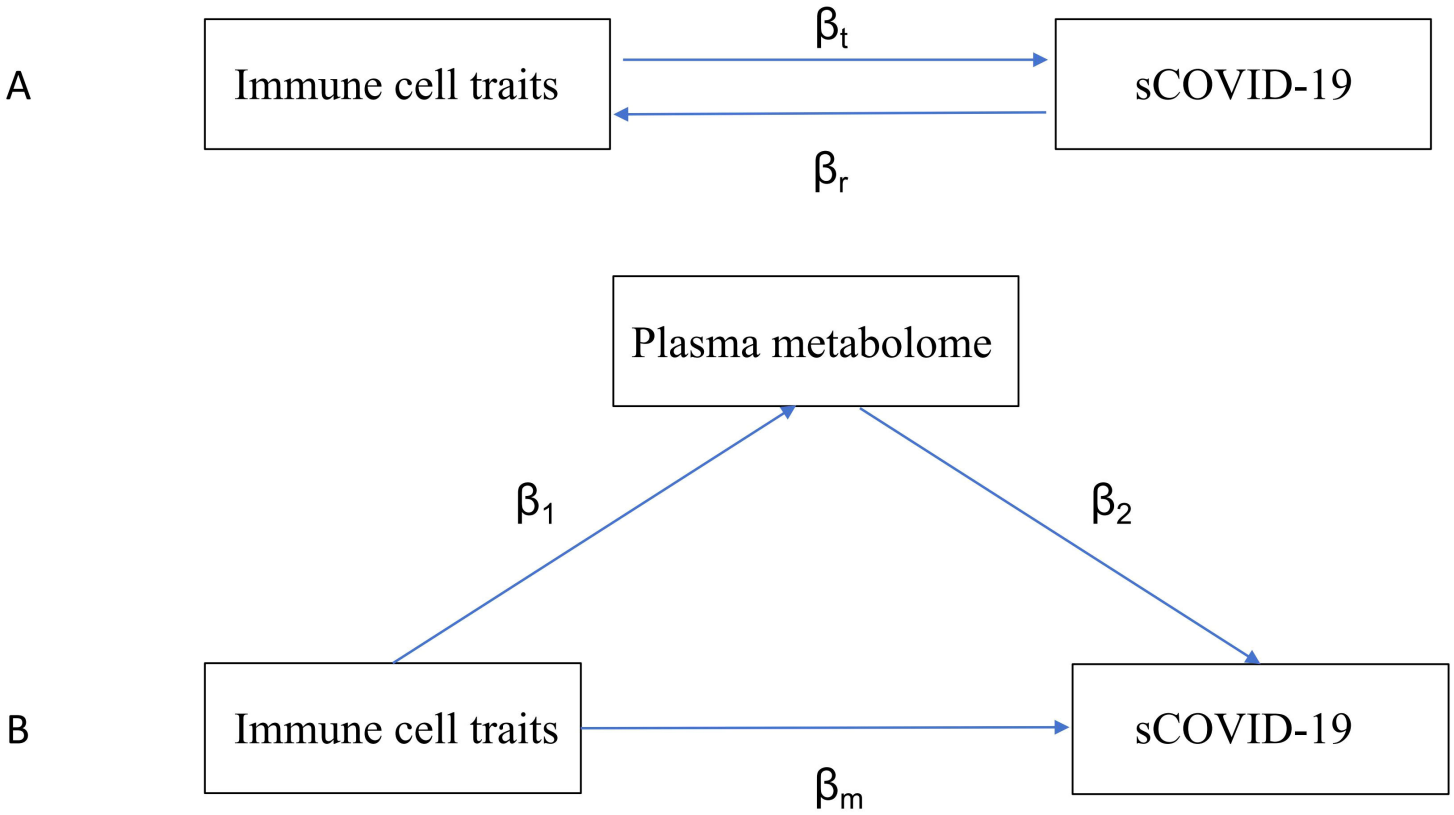
Diagrams illustrating associations examined in this study. **(A)** The total effect between immune traits and sCOVID-19. βt is the total effect using genetically predicted immune traits as exposure and sCOVID-19 as outcome. βr is the total effect using genetically predicted sCOVID-19 as exposure and immune traits as an outcome. **(B)** The total effect was decomposed into **(i)** indirect effect using a two-step approach (where β1 is the total effect of immune traits on plasma metabolome, and β2 is the effect of plasma metabolome on sCOVID-19) and the product method (β1 × β2) and **(ii)** direct effect (βm= βt -β1 × β2). Proportion mediated was the indirect effect divided by the total effect.

To explore mutual causality between immune phenotypes and sCOVID-19 risk (**Figure 1A**), we carried out a bidirectional 2S-MR assessment (i.e. total effect analysis). We achieved this by producing MR estimates using the inverse-variance weighted (IVW) approach. All SNPs are assumed to be genuine IVs in the IVW application. Thus, by employing this method, we obtained accurate estimation data. Then, as supplemental studies, we used the remaining 4 techniques (weighted median, weighted models, MR-Egger, and basic analyses) to verify the causal relationship between exposure factors and patient outcomes. This allowed us to validate the reliability of our results further (Li et al., 2023).

Mediation analysis is a statistical technique investigating how a factor influences the relationship between two other variables. In this study, we performed a mediation assessment using a 2S-MR analysis to determine if the plasma metabolome could mediate the causal connection between specific ICTs and the risk of sCOVID-19. The total immune cell influences on sCOVID-19 were categorized as follows: 1) direct influences of immune cell subsets on sCOVID-19 risk (βm in **Figure 1B**); and 2) indirect influences modulated by a mediator (β1×β2 in **Figure 1B**). Moreover, we computed the percentage regulated by the mediation by dividing the indirect effect by the total effect (Carter et al., 2021). Alongside, 95% confidence intervals were computed utilizing the delta formula.

### Sensitivity assessment

Using the MR-PRESSO distortion test, we tested alterations in the estimates made before and following outlier correction, and a p-value < 0.05 was set as the significance threshold. Outliers were eliminated, and the MR causal estimates were re-assessed. Cochran’s Q statistic and associated p values were employed to evaluate heterogeneity among selected IVs. After rejecting the null hypothesis, the random effects inverse variance weighting (IVW) method was utilized. The p-value for the MR-Egger intercept was calculated using directional pleiotropy. Finally, a leave-one-out analysis was conducted to validate the impact of particular SNPs on the overall causal estimates.

### Statistical analysis

The MR analyses were conducted using R version 4.2.1 and the “2S-MR” package (version 0.5.8). The MR-Pleiotropy Residual Sum and Outlier (MR-PRESSO) analysis was conducted using the “MRPRESSO” package in R programming language. We conducted a PhenoScanner analysis to assess all documented ICTs linked to our genes of interest. The significance threshold was set at a p.adjust value of less than 0.05.

## Results

### Causal relation between ICTs and sCOVID-19 risk

**Figure 2:**
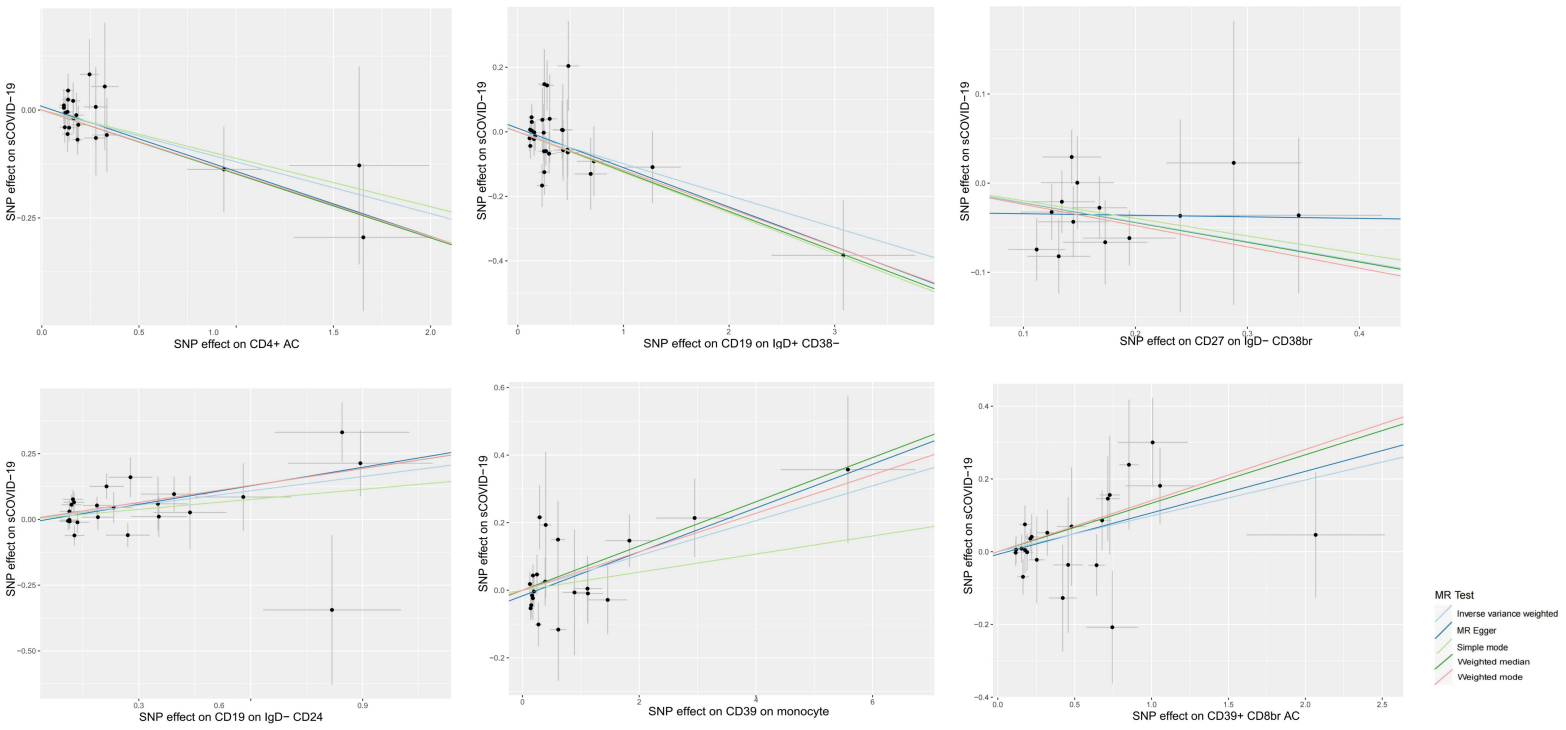
Scatter plots of MR analyses of causal effects for immune traits on the risk of sCOVID-19.

We used a 2S-MR analysis to identify the causal relationship between ICTs and sCOVID-19. Following careful screening, we found 18621 SNPs as IV (**Supplementary File 1**). Based on our F statistical analysis, there was no weak instrumental bias. At the significance of 0.01, our IVW analysis revealed a strong causal link between six ICTs and sCOVID-19 risk. Among these, three ICTs were casually linked to reduced sCOVID-19 risk, e.g., CD27 on IgD- CD38br (*OR* 0.804, *95% CI* 0.699–0.025, p = 0.002), CD4+ AC (*OR* 0.887, *95% CI* 0.812–0.969, p = 0.008), CD19 on IgD+ CD38- (*OR* 0.906, *95% CI* 0.849–0.967, p = 0.003). In contrast, three ICTs were casually associated with enhanced sCOVID-19 risk, e.g., CD39+ CD8br AC (*OR* 1.104, *95% CI* 1.028–1.185, p = 0.007), CD19 on IgD- CD24- (*OR* 1.199, *95% CI* 1.075–1.337, p = 0.001) and CD39 on monocyte (*OR* 1.053, *95% CI* 1.013–1.094, p = 0.009). The aforementioned causal links were supported by most of the remaining 4 MR analyses (**Figure 2**; **Supplementary File 2**).

Additionally, we revealed that the MR assessment data exhibited no reverse causality for genetically predicted sCOVID-19 risk concerning the six immune profiles (**Supplementary File 3**).

### The link between plasma metabolome and sCOVID-19 risk

**Figure 3:**
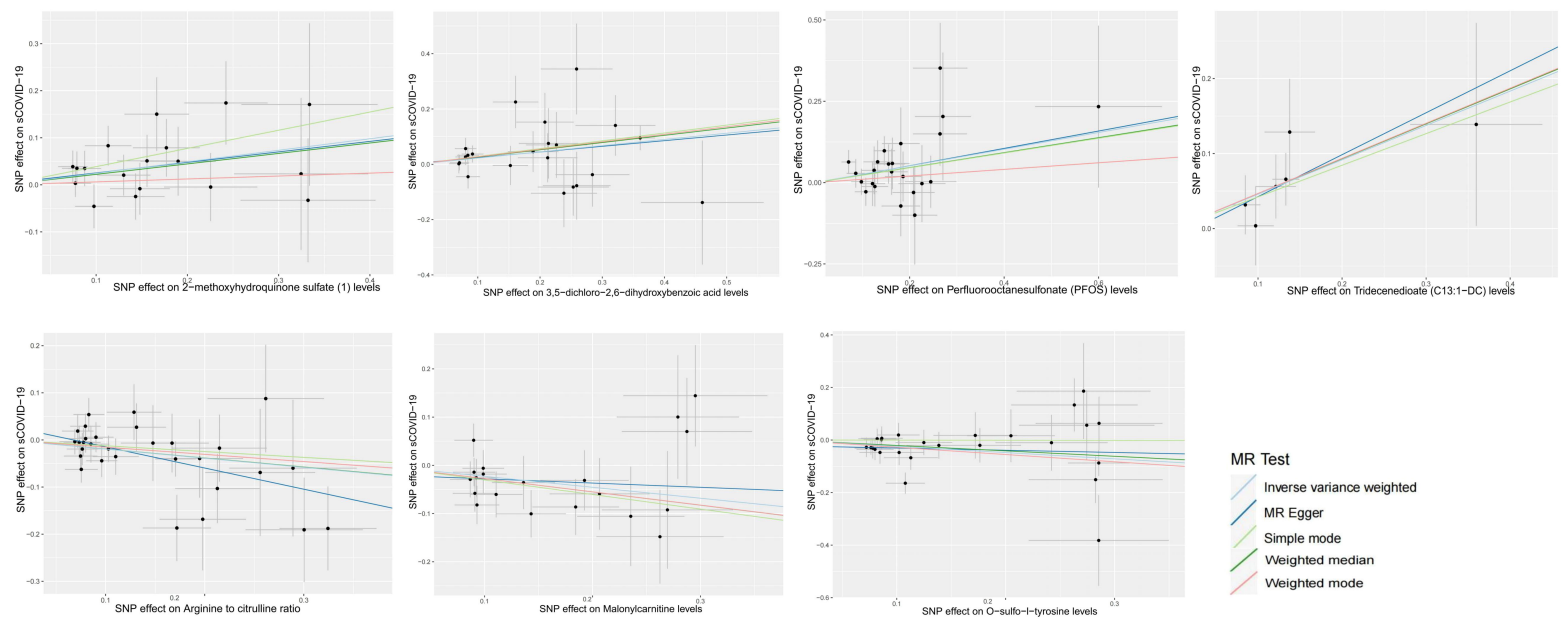
Scatter plots of MR analyses of causal effects for plasma metabolites on the risk of sCOVID-19.

Using IVM, 34843 SNPs were identified as IVs (**Supplementary File 4**), and seven PMs were strongly associated with sCOVID-19 risk at a significance level of 0.01. Among them, three PMs, namely, malonyl carnitine contents (*OR* 0.797, *95% CI* 0.672–0.945, p = 0.009), o-sulfo-l-tyrosine contents (*OR* 0.788, *95% CI* 0.664–0.934, p = 0.006) and arginine to citrulline ratio (*OR* 0.824, *95% CI* 0.721–0.942, p = 0.005) were casually related to reduced sCOVID-19 risk. Alternately, four PMs, namely, tridecenedioate (C13:1-DC) (*OR* 1.581, *95% CI* 1.167–2.143, p = 0.003), perfluorooctanesulfonate (PFOS) (*OR* 1.293, *95% CI* 1.077–1.552, p = 0.006), 3,5-dichloro-2,6-dihydroxybenzoic acid (*OR* 1.252, *95% CI* 1.080–1.451, p = 0.003) and 2-methoxyhydroquinone sulfate (1) (*OR* 1.278, *95% CI* 1.067–1.530, p = 0.008) were casually related to enhanced sCOVID-19 risk (**Figure 3**, **Supplementary File 5**).

### The link between ICTs and plasma metabolome

**Figure 4:**
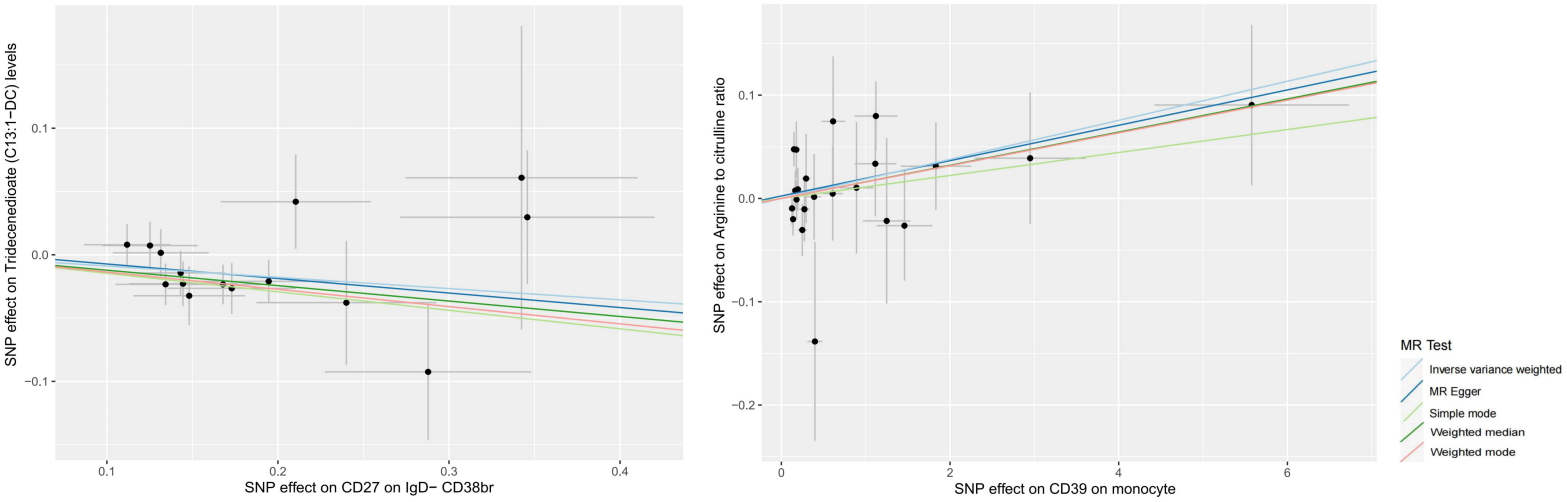
Scatter plots of MR analyses of causal effects for immune traits on plasma metabolites.

**Figure 5:**
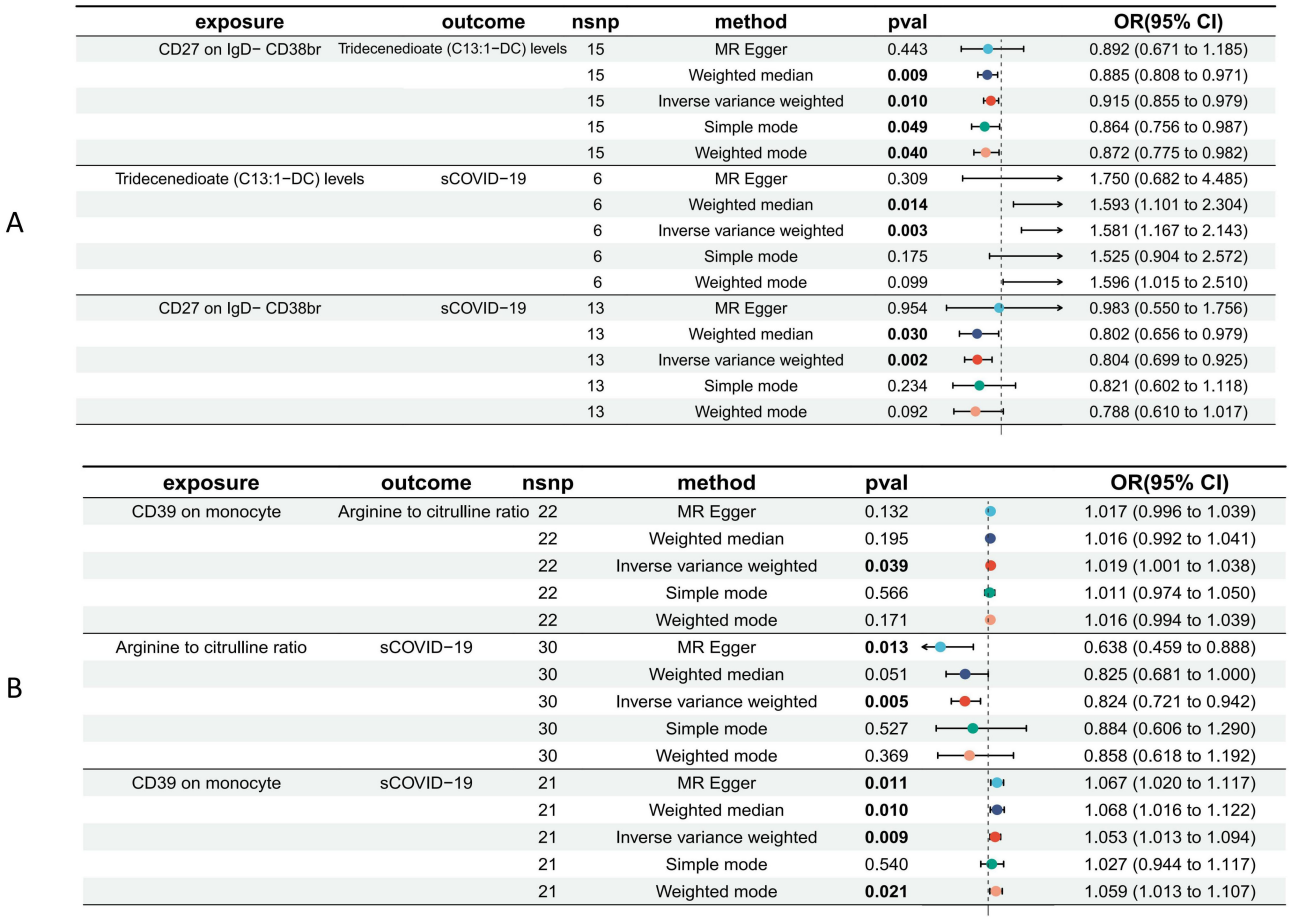
Forest plot to visualize the causal effects of Tridecenedioate (C13:1-DC) **(A)**, and arginine to citrulline ratio **(B)** with immune traits and sCOVID-19.

According to our MR analysis, there was a casual correlation between Tridecenedioate (*C13*:1-DC) levels and CD27 in IgD-CD38br (*OR* 0.915, 95% CI 0.855–0.979, p = 0.010) and a casual correlation between arginine to citrulline ratio and CD39 on monocyte (*OR* 1.019, 95% *CI* 1.001–1.038, p = 0.039) (**Figures 4**, **5**, **Supplementary File 6**). The genome-wide marked SNPs (i.e., IVs) are listed in **Supplementary File 7**.

**Figure 6:**
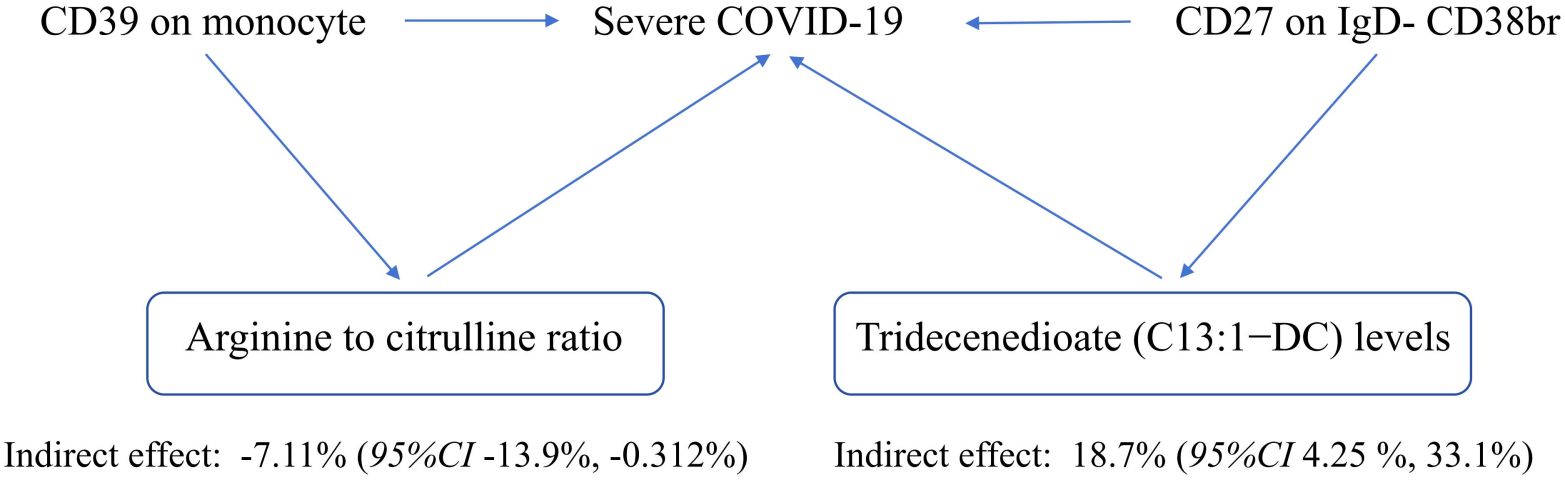
Schematic diagram of the effect of the immune traits mediation.

The plasma metabolome regulates the degree of association between immunological characteristics and the risk of sCOVID-19. We discovered a strong association between CD27 expression on IgD− CD38br cells and reduced levels of Tridecenedioate (C13:1−DC). This reduction in C13:1−DC was directly associated with an increased risk of severe COVID-19 infection. The Tridecenedioate (C13:1−DC) concentration made up 18.7% of the augmented sCOVID-19 risk linked to the CD27 on IgD− CD38br (proportion mediated: 18.7%; *95% CI* 4.25%, 33.1%). Arginine to citrulline ratio modulated -7.11% (*95% CI* -13.9%, -0.312%) CD39 action of monocyte on mediating sCOVID-19 risk (**Figures 5**, **6**).

### Sensitivity analysis

To evaluate the pleiotropy of causal estimates, we performed various sensitivity analyses. Using Cochran’s Q-test, we showed no heterogeneity in the causal relationship between the SNPs. We found no horizontal SNP multi-effect using the pleiotropy test (**Supplementary File 8**). We verified the effect of individual SNPs on total causal estimates using a leave-one-out analysis. Finally, we reran MR assessments for the remaining SNPs after removing the individual SNPs. Our findings were consistent, indicating that every SNP was calculated to obtain a statistically significant causal relationship (**Supplementary File 9**).

## Discussion

After thoroughly examining a substantial collection of publically accessible genetic data, we discovered that several immunophenotypes and PMs are closely associated with the likelihood of contracting sCOVID-19. Furthermore, we have discovered two immunological characteristics that can genetically forecast the risk of severe COVID-19. These qualities are partially influenced by two specific genetic markers known as PMs.

Upon COVID-19 infection, immuno- and inflammatory responses critically regulate the course of infection (Boechat et al., 2021; Merad et al., 2022; Primorac et al., 2022). Currently, sCOVID-19 has been associated with significant changes in immune activity outside the central immune system. These changes include increased innate immunological or inflammatory responses and reduced adaptive immune response. CD4+ and CD8+ T cells elicited by SARS-CoV-2 infection are directed against various antigens and are significantly associated with milder disease. Extensive lymphopenia (involving CD4+ AC) is potentially modulated by lymphocyte sequestering within tissues or proinflammatory cytokine-induced apoptosis and may contribute to defective viral clearance (Boechat et al., 2021). Coinhibitory receptors on T cells (e.g., CD8 on CD39+ CD8br) recognizing SARS-CoV-2 peptide pools were associated with increased frequencies of cytokine-producing T cells, contributing to enhanced disease severity (Shahbaz et al., 2021b). A large number of plasmablast or mature B cells (e.g., CD19 on IgD- CD24-) expansion (reaching 30% of serum B cells and with some association with extrafollicular responses) are also found in sCOVID-19 patients (Mansourabadi et al., 2023). The high plasmablast population may indicate poly-reactivity as there are relatively low frequencies of somatic mutations in antibody clones within patients, thereby eliciting reduced viral management and promoting tissue damage (De Biasi et al., 2020). The Memory B cells (MBCs) play a crucial role in controlling the occurrence and severity of COVID-19 infection. Several investigations have found that the number of switched (e.g., CD27 on IgD- CD38br) and unswitched (e.g., CD19 on IgD+ CD38-) memory B cells is significantly reduced in COVID-19 patients. This reduction is independently associated with higher severity and mortality rates in these patients (De Biasi et al., 2020; Colkesen et al., 2022; Primorac et al., 2022; Mansourabadi et al., 2023). During primary SARS-CoV-2 infection, T cells assist B cell differentiation in the germinal centres (GC) and recruit a large repertoire of MBCs. Patients with inadequate T cells have significantly poorer GC responses, lower antigen-specific antibodies, and fewer switched MBCs. As a result, they develop a more severe form of the disease (Sosa-Hernandez et al., 2020).

In sCOVID-19, monocyte activation and expansion result in hyperinflammation, which, in turn, causes capillary hyperpermeability, coagulation dysfunction and substantial tissue damage (Boechat et al., 2021; Primorac et al., 2022). CD39 is ubiquitously expressed in human peripheral blood on > 90% of monocytes (Diaz-Garcia et al., 2022). Wang et al. reported augmented CD39 expression within the lung, liver, spleen, and PBMCs of sCOVID-19 patients, which was intricately linked to the durations of hospital and intensive care unit (ICU) stays, as well as the markers of coagulation, suggesting strong links between ectonucleotidases and disease progression (Diaz-Garcia et al., 2022). Using bioinformatics, Schultz et al. revealed up-regulated CD39 contents within the leukocytes of COVID-19 patients (Schultz et al., 2022).

Emerging studies identified several PMs as strong modulators of the COVID-19 disease course. L-arginine regulates many biological processes, including COVID-19 (Rees et al., 2021; Reizine et al., 2021; Sacchi et al., 2021). Claudia Morris and colleagues reported that both COVID-19-infected adults and children exhibit markedly diminished plasma L-Arginine (as well as L-Arginine bioavailability) relative to controls (Rees et al., 2021). Reizin and his colleagues revealed strongly downregulated arginine concentration among 13 sCOVID-19 patients and 13 with moderate pneumonia relative to 13 healthy volunteers (Reizine et al., 2021). As previously stated, blood samples were collected during admission and on the fourth and seventh days of hospitalization. They identified the most significant arginine downregulation upon admission, i.e., 26% and 54% for mild and severe COVID-19, respectively, compared to healthy controls.

Interestingly, the T-cell quantity was linked strongly with arginine content and was similarly reduced in COVID-19 patients. Furthermore, a study of T-cell proliferative capacity revealed that COVID-19 patients had much lower T-cell proliferative ability, which may be restored with arginine supplements. In another study, Alessandra Sacchi et al (Sacchi et al., 2021). revealed that plasma L-Arginine content was inversely proportional to COVID-19 severity. They also revealed that the activated GPIIb/IIIa complex responsible for platelet activation and thromboembolic events was strongly elevated in platelets of sCOVID-19 patients. Previous studies have found that CD71+ erythroid cells (CECs) are expanded in COVID-19 patients, especially those with severe disease. These CECs express arginase I and II, which could be responsible for the reduced L-arginine levels in COVID-19 patients (Shahbaz et al., 2021a; Elahi, 2022; Saito et al., 2022).

L-arginine is a substrate for numerous enzymatic reactions. Its metabolism utilizes 3 primary networks: (1) L-Arginine to L-ornithine (Arginase-mediated), (2) L-Arginine to agmatine (L-Arginine decarboxylase-mediated), and (3) L-Arginine to NO and citrulline (nitric oxide (NO) synthase (NOS)-mediated) (Adebayo et al., 2021). NO demonstrates both indirect and direct antiviral effects. Direct inhibition of NO can effectively decrease viral activity. Therefore, NO is considered one of the early host reactions against viruses.

In contrast, the indirect NO effects include inflammatory and immune response modulation. NO accelerates several reactive oxygen and nitrogen species formation, synergistically opposing viral activity (Adebayo et al., 2021). During the acute phase of COVID-19, there is a large increase in arginase activity. This leads to malfunctioning the immune system and blood vessels, inflammation, and blood clot formation. This is caused by a decrease in the concentration of L-arginine in the blood and a shift in metabolism that reduces nitric oxide production. Acute COVID-19 is also correlated with diminished plasma L-arginine contents, which, in turn, modulates myeloid suppressor cell growth and decreases T-cell proliferation, two typical inflammatory features of severe disease (Zhu et al., 2014). Arginine also induces CD4+ and CD8+ T cell survival by switching metabolism to oxygen consumption, increasing free respiratory capacity in activated T cells (Bronte and Zanovello, 2005). Human monocytes consume significant amounts of glutamine and maintain adequate enzymatic activity to convert glutamine to citrulline and, subsequently, citrulline to arginine. This process decreases the ratio of arginine to citrulline, which supports our research findings (Derakhshani et al., 2021).

Perfluorooctanesulfonic acid (PFOS) belongs to Per- and polyfluoroalkyl substances (PFAS), a class of artificial organic chemicals possessing both hydrophilic and hydrophobic properties (Gluge et al., 2020). PFAS is strongly associated with multiple health conditions, such as hepatotoxicity, dyslipidemia, endocrine outcomes, and immunotoxicity (Kvalem et al., 2020). Human epidemiological studies have shown that children’s blood antibody response following vaccination is adversely affected by PFOS exposure. Furthermore, exposure to PFOS during pregnancy may increase the risk of infection (Abraham et al., 2020). The National Toxicology Program concluded that PFOA and PFOS pose an immunologic hazard to humans due to their strong suppression of antibody response observed in animal and human studies (Kim et al., 2021). One Italian investigation reported augmented COVID-19-related mortality risk among people who received heavy exposure to PFAS (Catelan et al., 2021). It has been suggested that the probable cause of this was the powerful immunosuppressive effects of PFAS and the deposition of PFAS in lung tissue, either alone or in conjunction with pre-existing PFAS-related diseases, which hastened the course of COVID-19. Philippe Grandjean et al. found increased plasma-PFAS levels among Danish COVID-19 patients. This increase was strongly linked to a higher likelihood of needing intensive care or death (Grandjean et al., 2020). In the present study, we verified the critical connection between PFOS, immune cells, and their control over COVID-19. Still, we also proposed that PFOS is a strong regulator of the immunological characteristic and the correlation between COVID-19 and it. Previous research has demonstrated that methoxyphenolic compounds have anti-inflammatory characteristics in leukocytes, which could potentially provide some degree of protection in a hyperinflammatory condition (Mrityunjaya et al., 2020; Perez de la Lastra et al., 2023). However, we found that 2−methoxyhydroquinone sulfate directly increased the risk of sCOVID-19. However, the fundamental mechanism still needs to be understood.

Our research limitations are: Firstly, this investigation was conducted on a purely European population. Therefore, the conclusions cannot be generalized to the worldwide population. Secondly, although we analyzed the largest COVID-19 GWAS summary statistics to date, the data comprised meta-analyses from numerous investigations. As a result, the sample could be diverse, indicating that the baseline clinical characteristics, patient demographics, distribution of concomitant diseases,recruitment time, vaccination status and other factors may have differed among different groups. In addition, the control population was not vetted, which means that there is a possibility that individuals without symptoms or with minor symptoms were unintentionally included in the control group. Thirdly, despite our attempts to identify and eliminate outlier variants, we could not fully exclude the potential of horizontal pleiotropy affecting our conclusions. Fourthly, we employed summary-level statistics and not individual-level data. Hence, we could not further examine causal relationships between various subcategories, including patient sex, race, diet, and disease status. Finally, no subsequent inquiries were conducted to verify the established cause-and-effect relationships in this inquiry. Therefore, conducting thorough mechanistic investigations to confirm the relationships mentioned above is crucial.

## Conclusions

In conclusion, our extensive MR analyses identified multiple ICTs and PMs that exhibited strong causal links to sCOVID-19 risk. Furthermore, we found two mediated relationships between the identified PMs and ICTs. For COVID-19 patient risk classification and outcome calculation, the ICTs and PMs—which were previously mentioned—are, therefore, promising bioindicator candidates. They can also significantly support investigating the underlying mechanisms governing Pathology and infection with sCOVID-19.

## Data Availability

The original contributions presented in the study are included in the article/**Supplementary Material**. Further inquiries can be directed to the corresponding author.

## Supplementary material

Supplementary File 1 Characteristics of significant SNPs with genome-wide associations for immune cells on sCOVID-19.

Supplementary File 2 MR analysis results of the effect of immune cells on sCOVID-19.

Supplementary File 3 Reverse MR analysis results of the effect of sCOVID-19 on immune cells.

Supplementary Fle 4 Characteristics of significant SNPs with genome-wide associations for plasma metabolites on sCOVID-19.

Supplementary File 5 MR analysis results of the effect of plasma metabolites on sCOVID-19.

Supplementary File 6 MR analysis results of the effect of immune cells on plasma metabolites.

Supplementary File 7 Characteristics of significant SNPs with genome-wide associations for immune cells on plasma metabolites.

Supplementary File 8 Heterogeneity and pleiotropy in MR analyses.

Supplementary File 9 Leave-one-out and Funnel plots to visualize the overall heterogeneity of MR analyses
