# Supplementary material for: Identifying the plasma metabolome responsible for mediating immune cell action in severe COVID-19: a Mendelian randomization investigation
Source: Front Cell Infect Microbiol. 2024 Aug 19;14:1393432. doi: 10.3389/fcimb.2024.1393432 (PMC11366714; doi:10.3389/fcimb.2024.1393432)

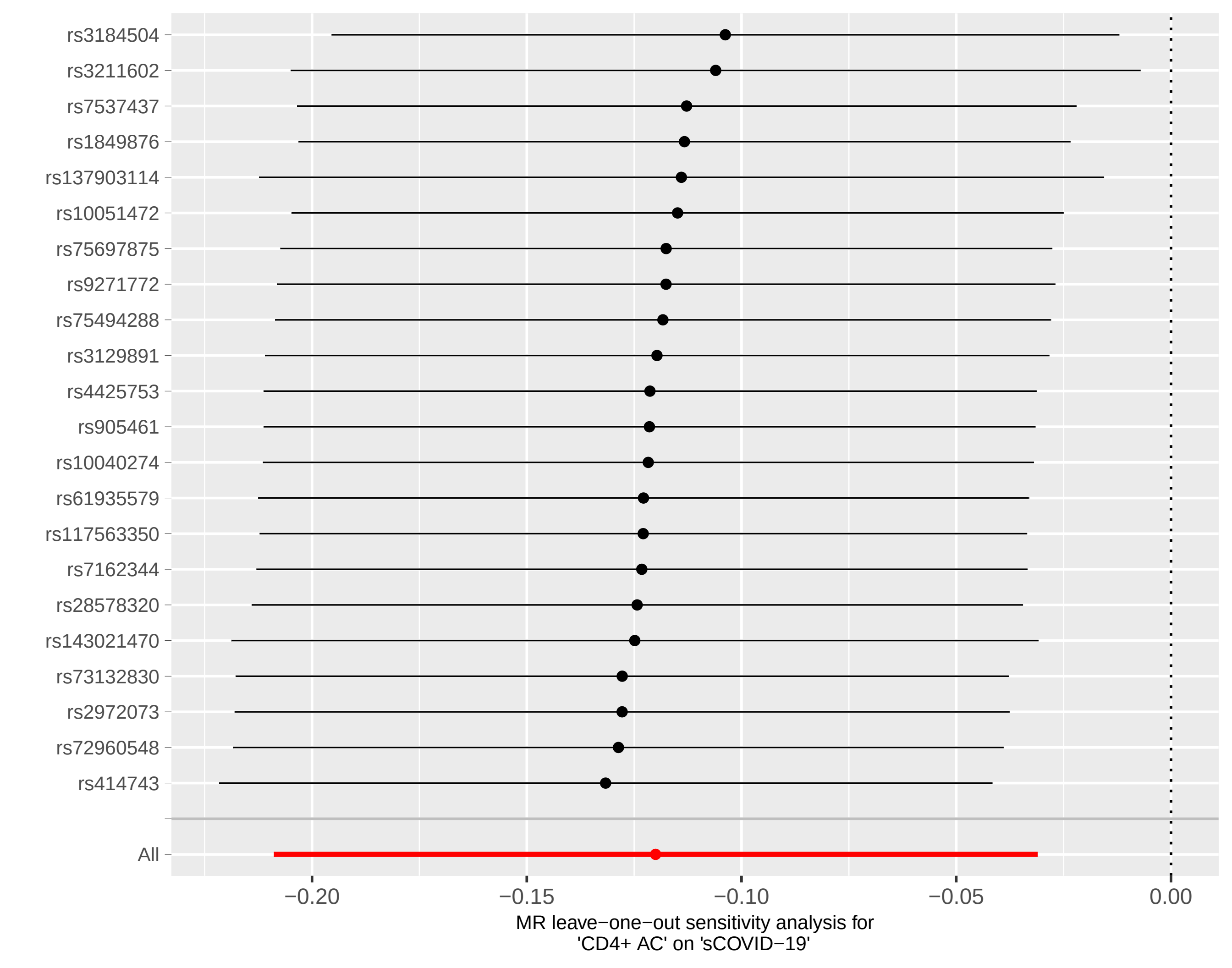
rs3184504
rs3211602
rs7537437
rs1849876
rs137903114
rs10051472
rs75697875
rs9271772
rs75494288
rs3129891
rs4425753
rs905461
rs10040274
rs61935579
rs117563350
rs7162344
rs28578320
rs143021470
rs73132830
rs2972073
rs72960548
rs414743
All
−0.20
−0.15
−0.10
−0.05
0.00
MR leave−one−out sensitivity analysis for
'CD4+ AC' on 'sCOVID−19'

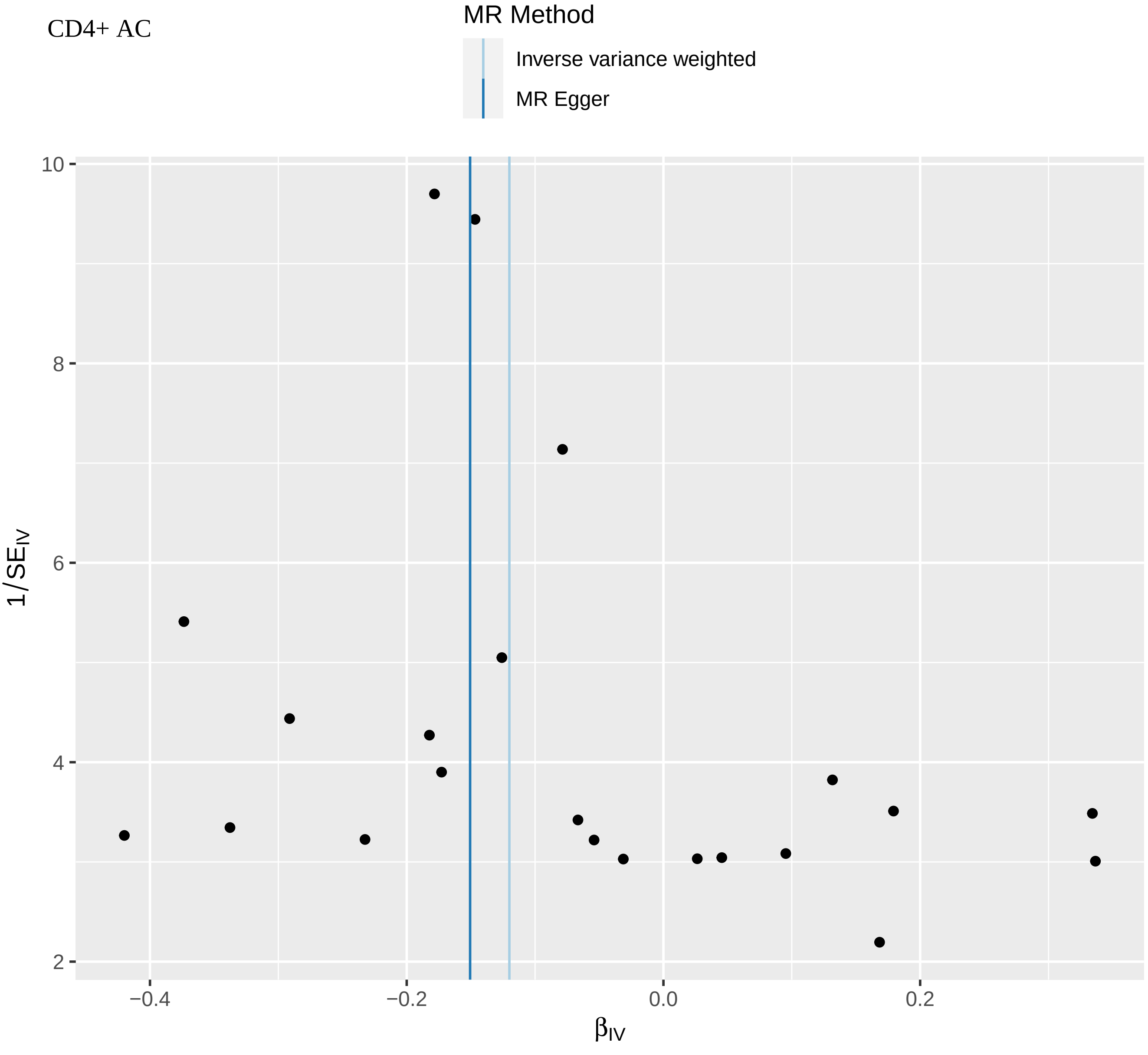
CD4+ AC
MR Method
Inverse variance weighted
MR Egger
10
8
6
4
2
1/SE_IV
−0.4
−0.2
0.0
0.2
β_IV

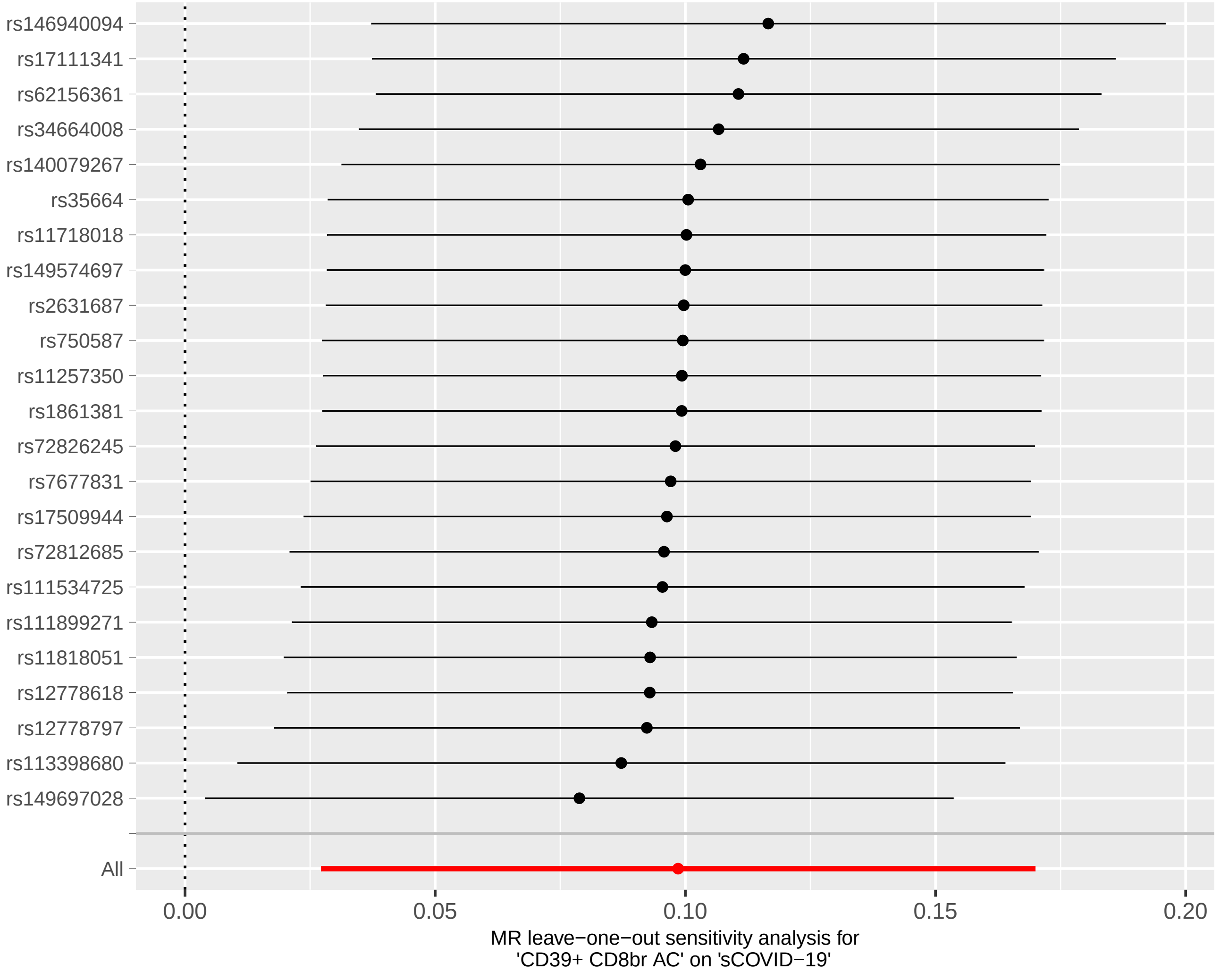
rs146940094
rs17111341
rs62156361
rs34664008
rs140079267
rs35664
rs11718018
rs149574697
rs2631687
rs750587
rs11257350
rs1861381
rs72826245
rs7677831
rs17509944
rs72812685
rs111534725
rs111899271
rs11818051
rs12778618
rs12778797
rs113398680
rs149697028
All
0.00
0.05
0.10
0.15
0.20
MR leave−one−out sensitivity analysis for
'CD39+ CD8br AC' on 'sCOVID−19'

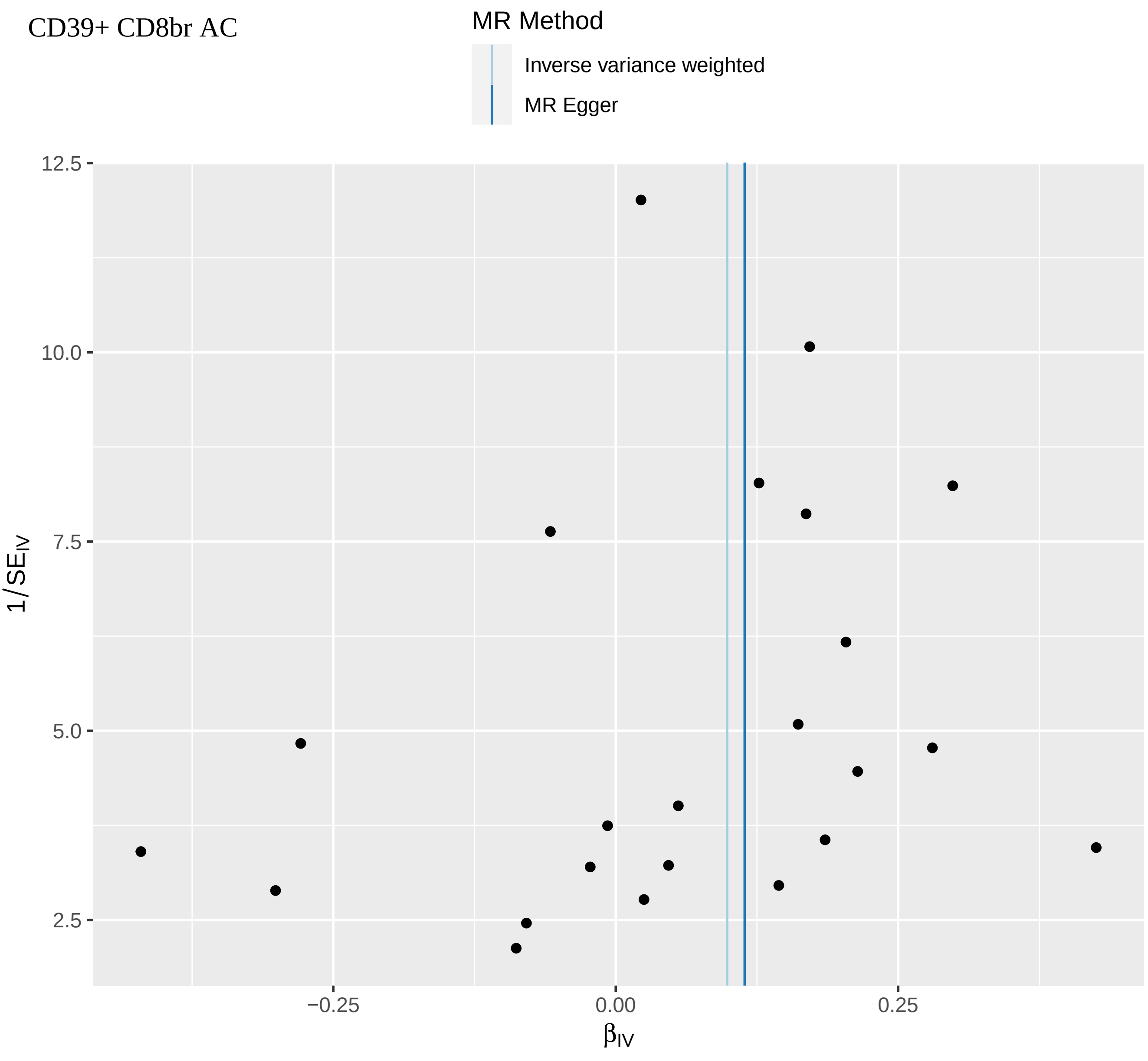
CD39+ CD8br AC
MR Method
Inverse variance weighted
MR Egger
12.5
10.0
7.5
5.0
2.5
1/SE_IV
−0.25
0.00
0.25
β_IV

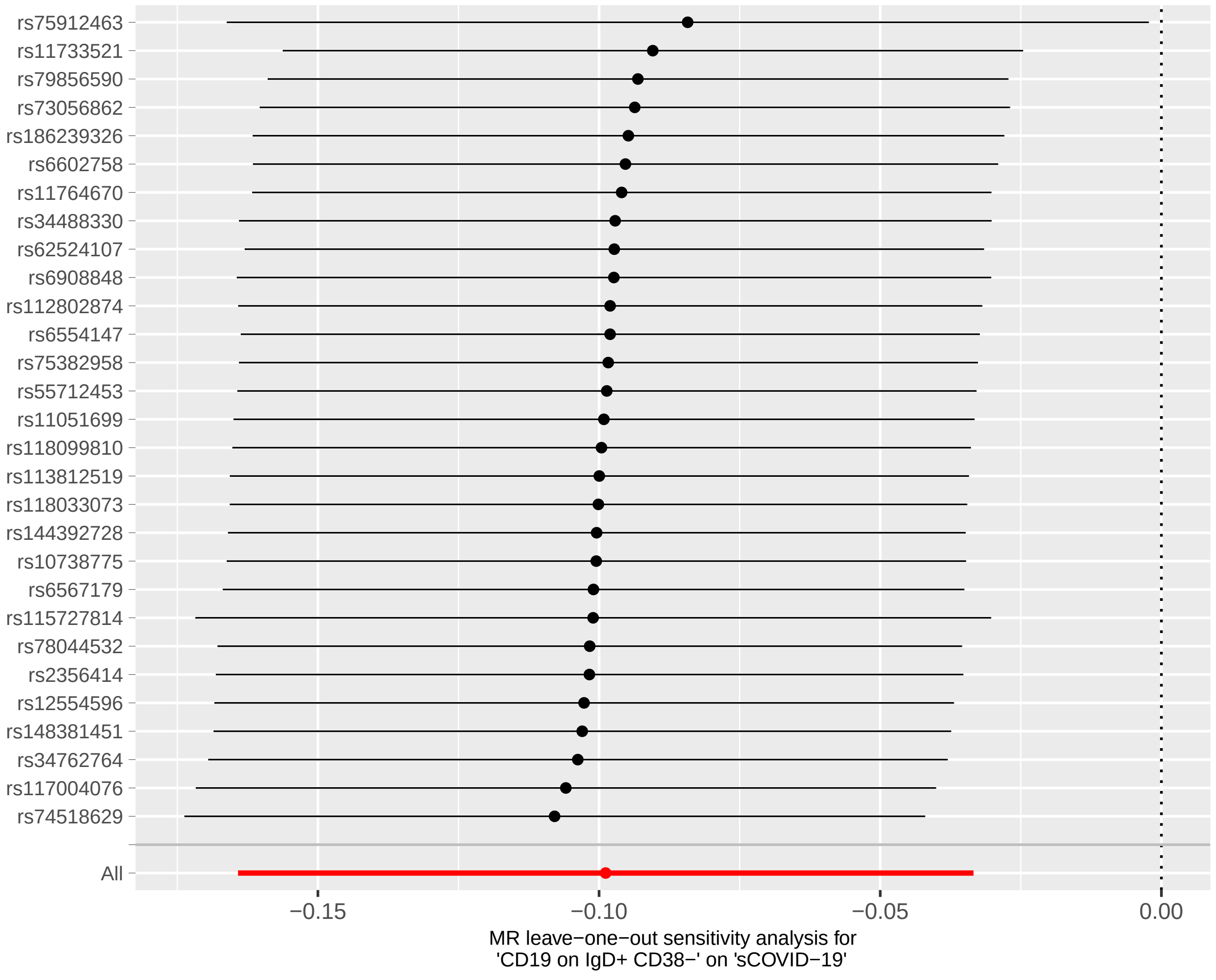
rs75912463
rs11733521
rs79856590
rs73056862
rs186239326
rs6602758
rs11764670
rs34488330
rs62524107
rs6908848
rs112802874
rs6554147
rs75382958
rs55712453
rs11051699
rs118099810
rs113812519
rs118033073
rs144392728
rs10738775
rs6567179
rs115727814
rs78044532
rs2356414
rs12554596
rs148381451
rs34762764
rs117004076
rs74518629
All
−0.15
−0.10
−0.05
0.00
MR leave−one−out sensitivity analysis for
'CD19 on IgD+ CD38−' on 'sCOVID−19'

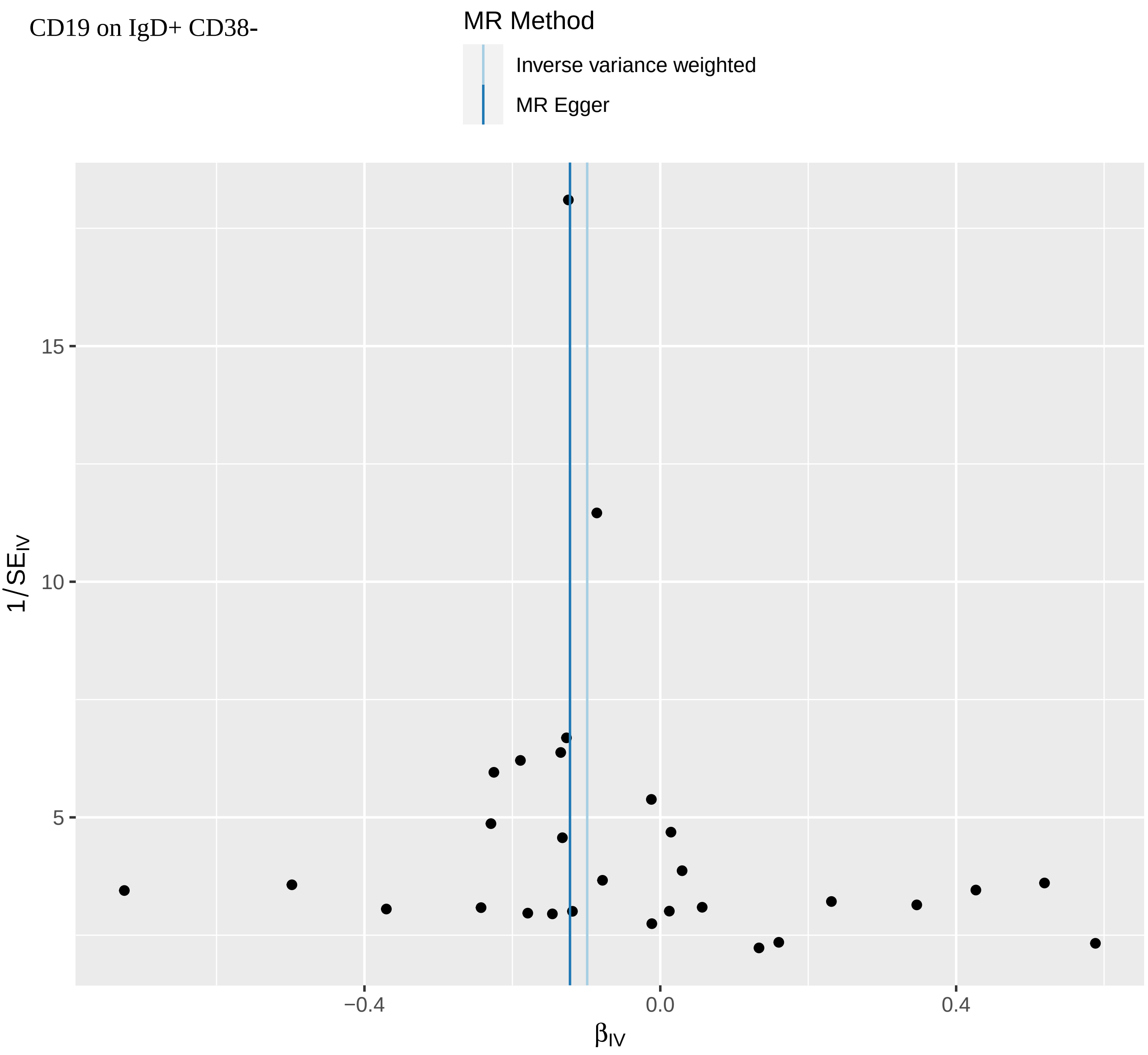
CD19 on IgD+ CD38-
MR Method
Inverse variance weighted
MR Egger
15
10
5
1/SE_IV
−0.4
0.0
0.4
β_IV

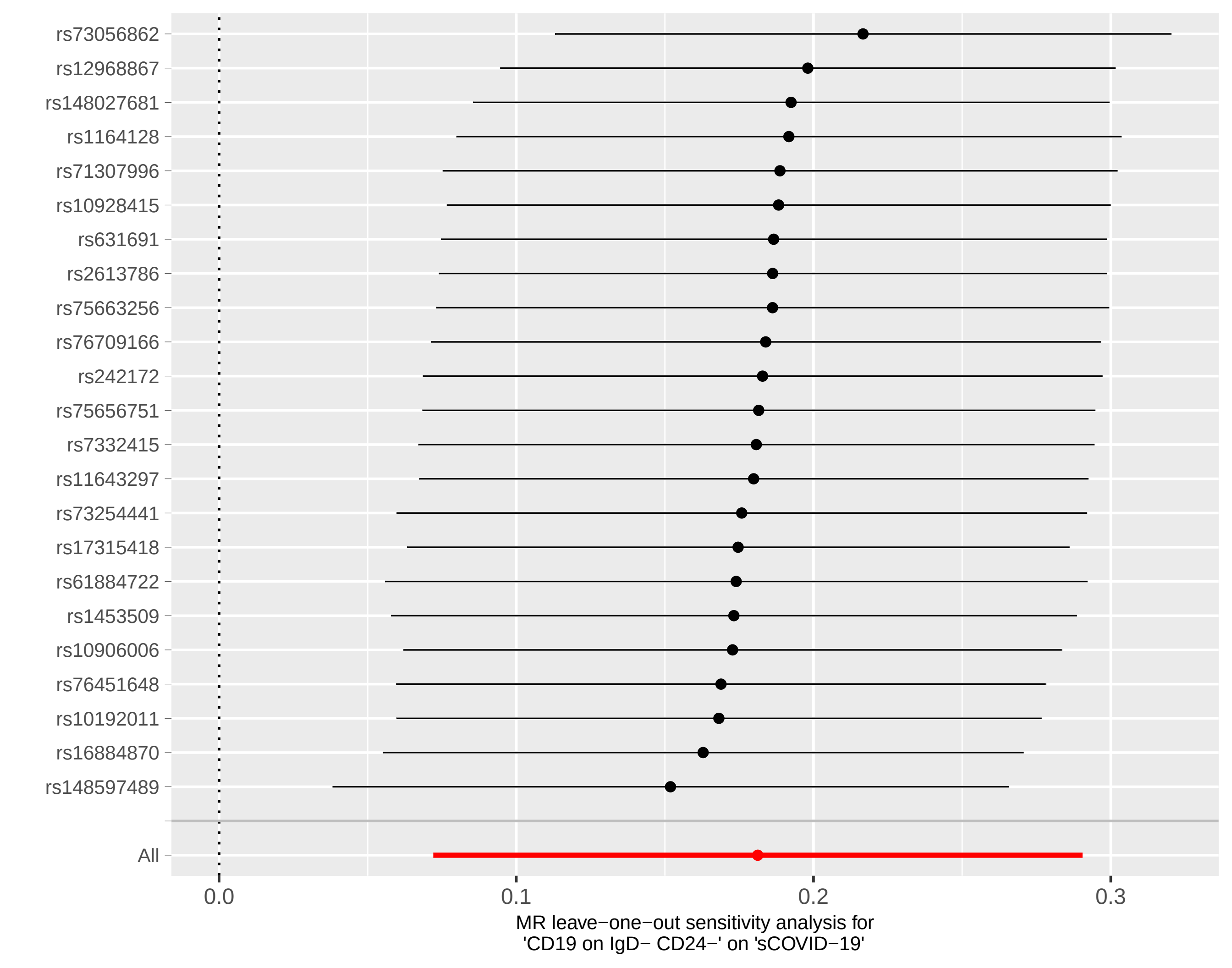
rs73056862
rs12968867
rs148027681
rs1164128
rs71307996
rs10928415
rs631691
rs2613786
rs75663256
rs76709166
rs242172
rs75656751
rs7332415
rs11643297
rs73254441
rs17315418
rs61884722
rs1453509
rs10906006
rs76451648
rs10192011
rs16884870
rs148597489
All
0.0
0.1
0.2
0.3
MR leave−one−out sensitivity analysis for
'CD19 on IgD− CD24−' on 'sCOVID−19'

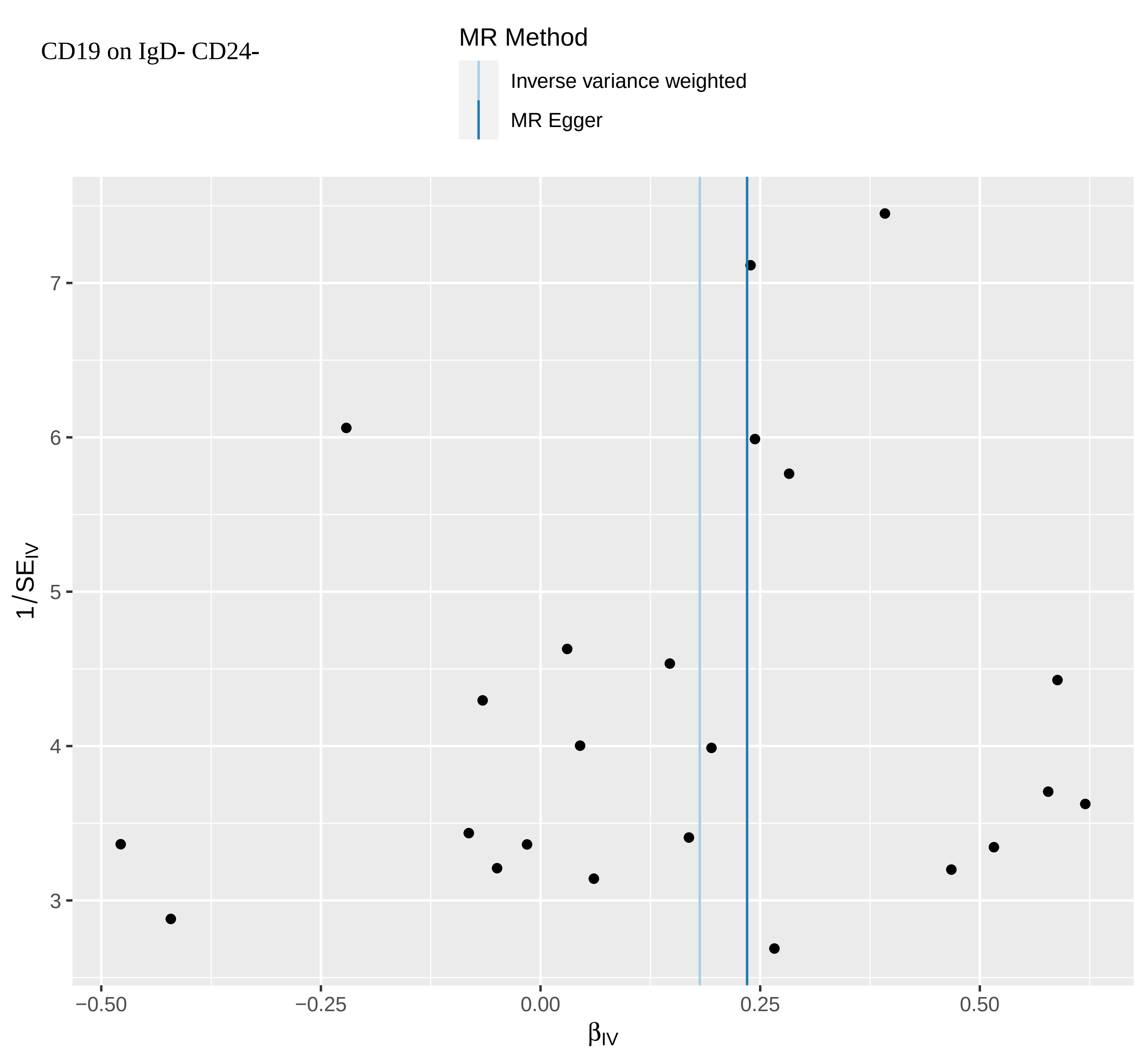
CD19 on IgD- CD24-
MR Method
Inverse variance weighted
MR Egger
1/SE_IV
β_IV
7
6
5
4
3
−0.50
−0.25
0.00
0.25
0.50

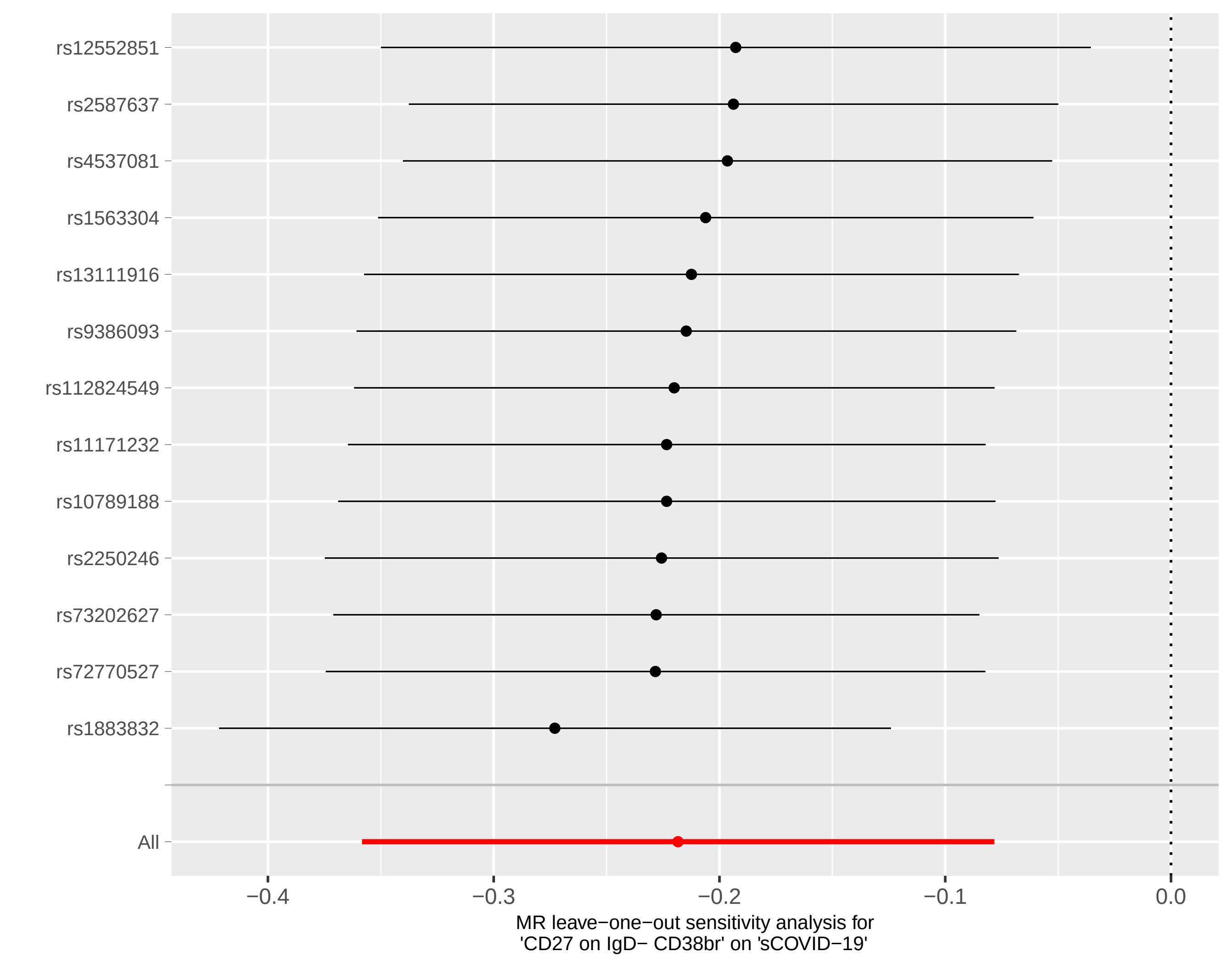
rs12552851
rs2587637
rs4537081
rs1563304
rs13111916
rs9386093
rs112824549
rs11171232
rs10789188
rs2250246
rs73202627
rs72770527
rs1883832
All
−0.4
−0.3
−0.2
−0.1
0.0
MR leave−one−out sensitivity analysis for
'CD27 on IgD− CD38br' on 'sCOVID−19'

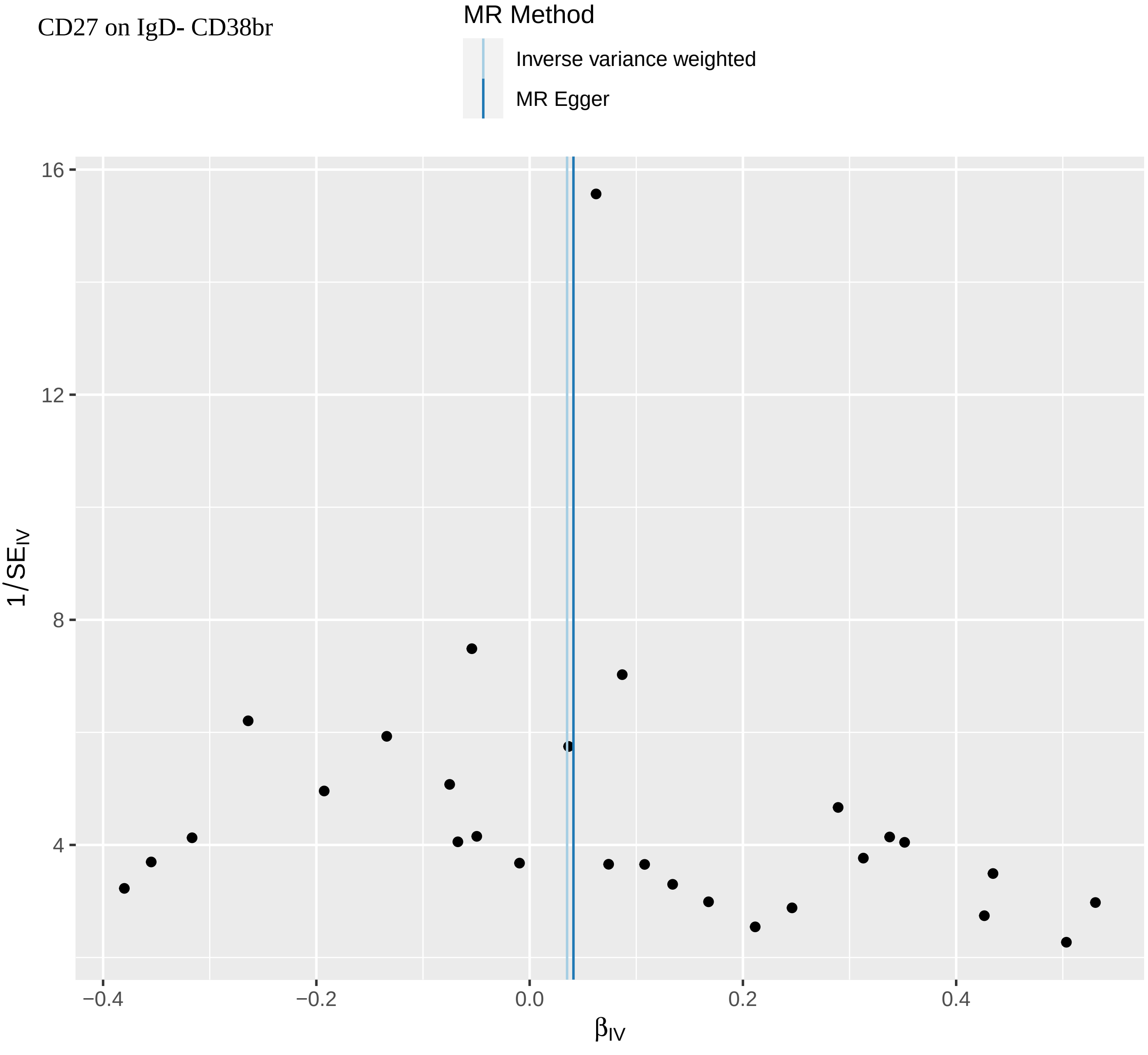
CD27 on IgD- CD38br
MR Method
Inverse variance weighted
MR Egger
16
12
8
4
1/SE_IV
−0.4
−0.2
0.0
0.2
0.4
β_IV

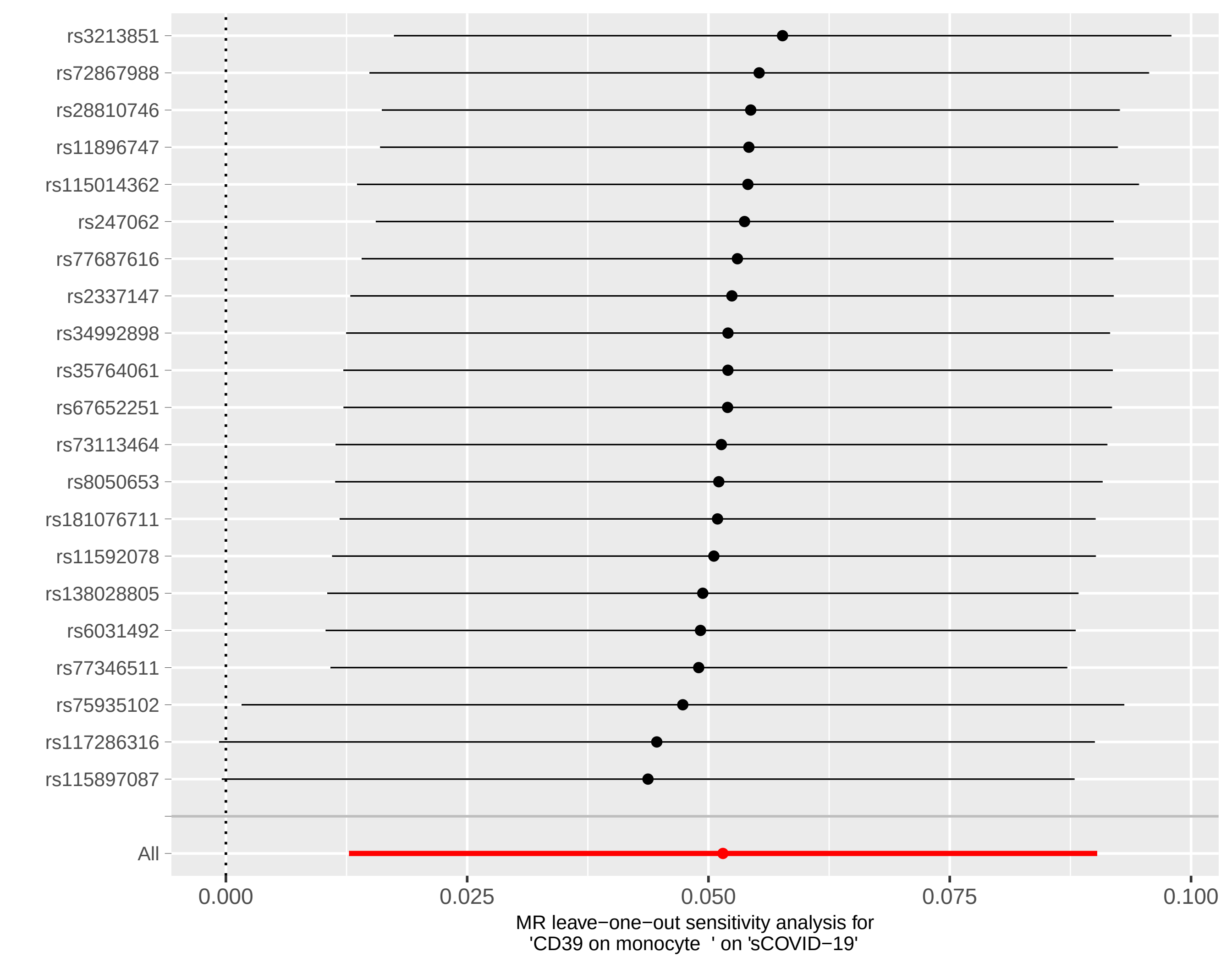
rs3213851
rs72867988
rs28810746
rs11896747
rs115014362
rs247062
rs77687616
rs2337147
rs34992898
rs35764061
rs67652251
rs73113464
rs8050653
rs181076711
rs11592078
rs138028805
rs6031492
rs77346511
rs75935102
rs117286316
rs115897087
All
0.000
0.025
0.050
0.075
0.100
MR leave−one−out sensitivity analysis for
'CD39 on monocyte ' on 'sCOVID−19'

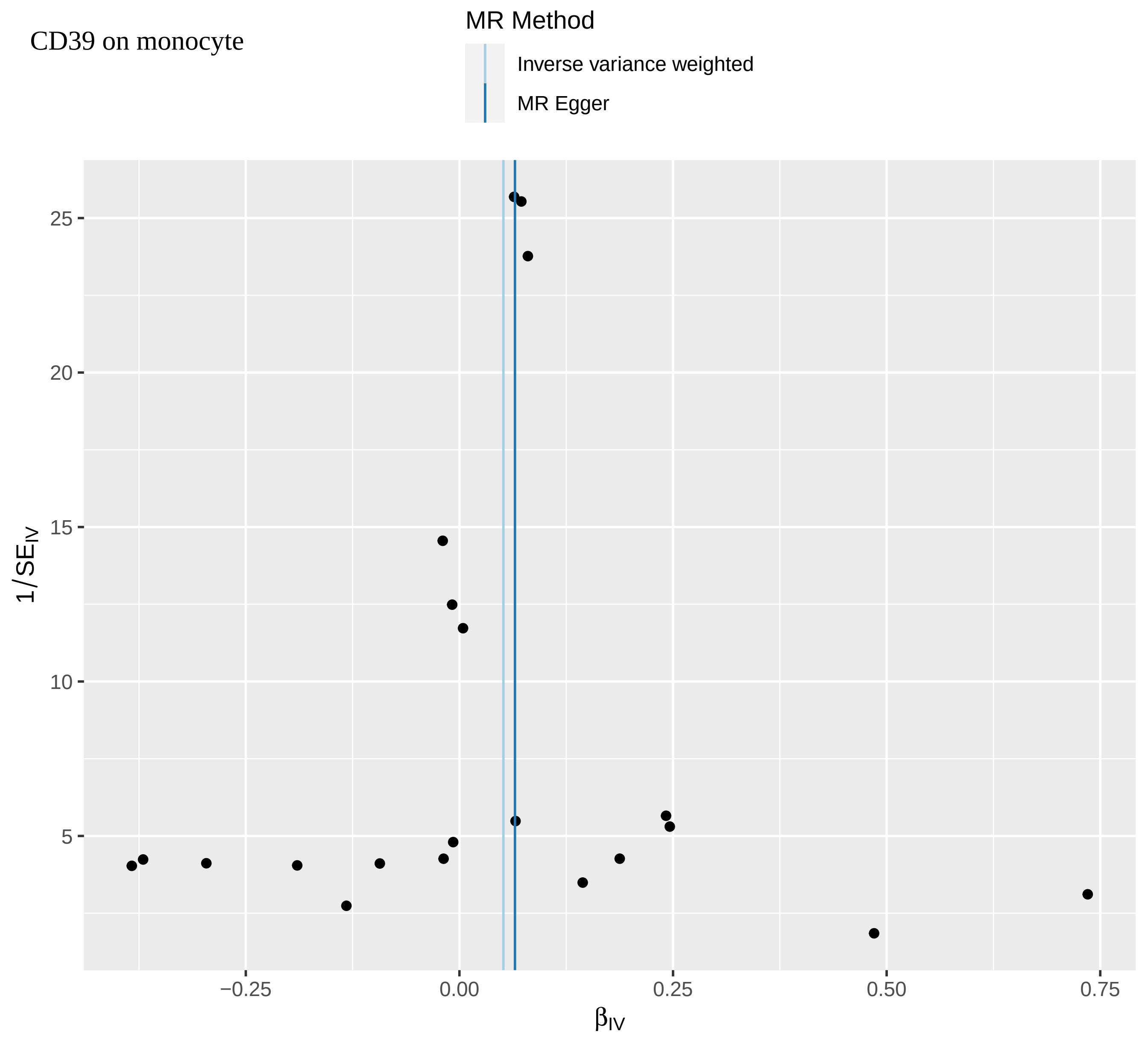
CD39 on monocyte
MR Method
Inverse variance weighted
MR Egger
25
20
15
10
5
1/SE_IV
−0.25
0.00
0.25
0.50
0.75
β_IV

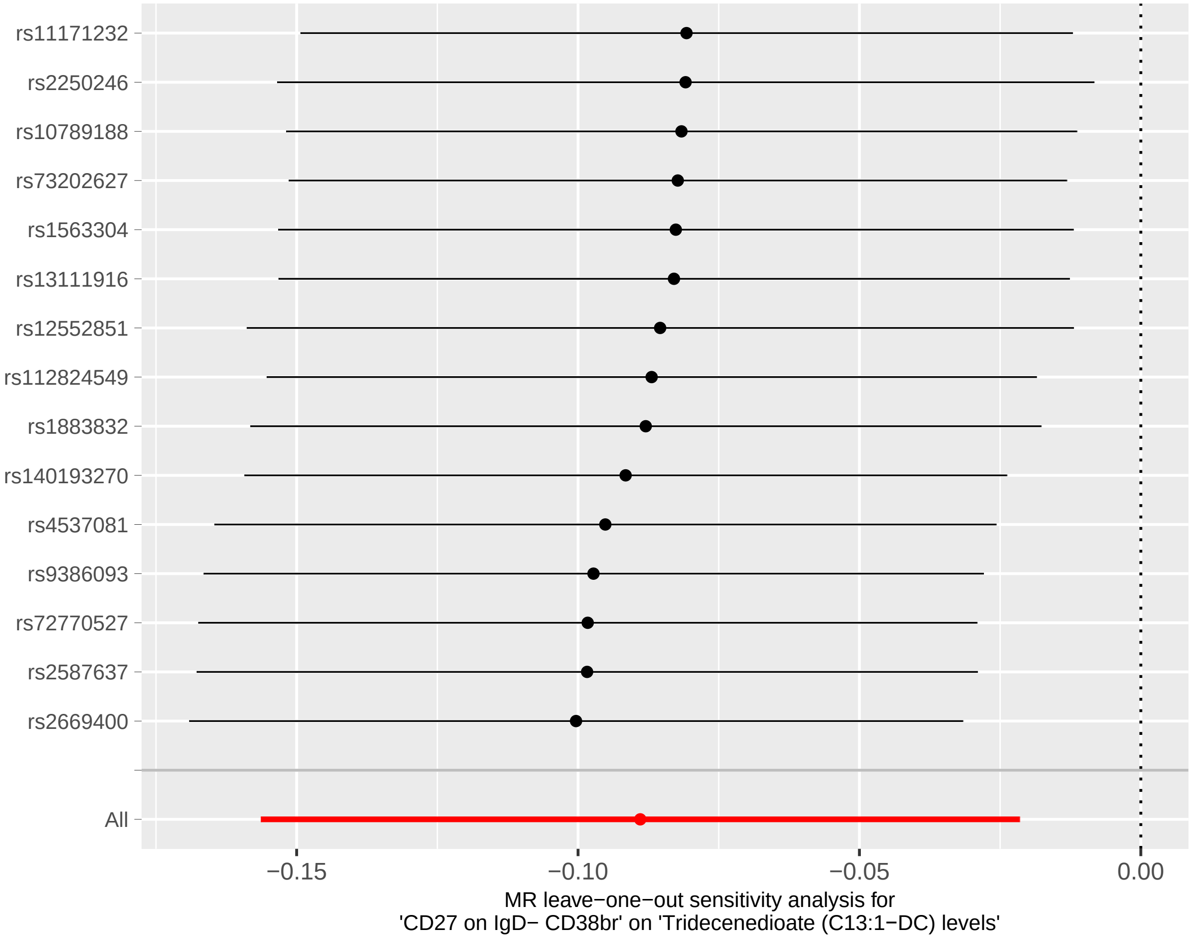
rs11171232
rs2250246
rs10789188
rs73202627
rs1563304
rs13111916
rs12552851
rs112824549
rs1883832
rs140193270
rs4537081
rs9386093
rs72770527
rs2587637
rs2669400
All
−0.15
−0.10
−0.05
0.00
MR leave−one−out sensitivity analysis for
'CD27 on IgD− CD38br' on 'Tridecenedioate (C13:1−DC) levels'

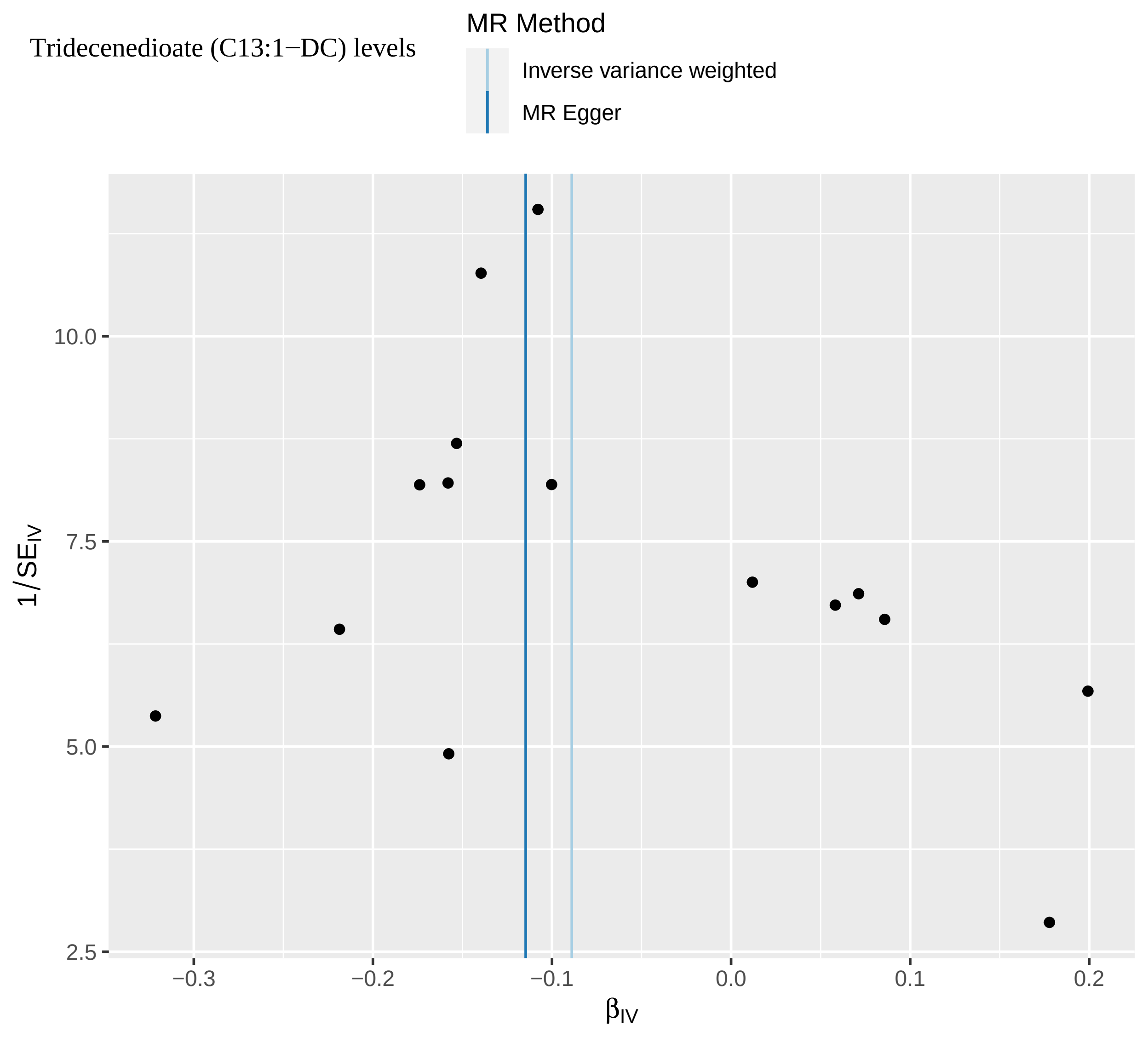
Tridecenedioate (C13:1−DC) levels
MR Method
Inverse variance weighted
MR Egger
10.0
7.5
5.0
2.5
1/SE$_{IV}$
−0.3
−0.2
−0.1
0.0
0.1
0.2
β_{IV}

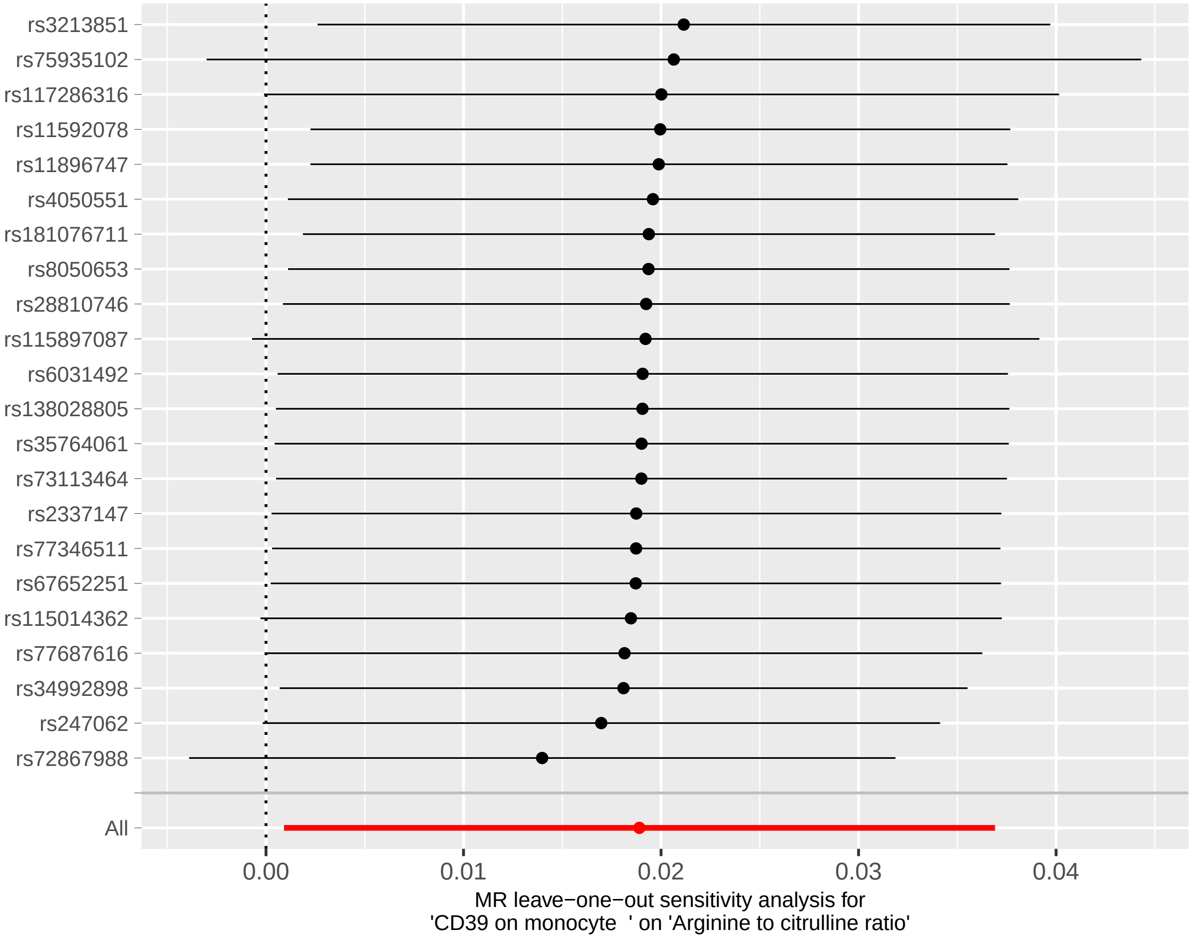
rs3213851
rs75935102
rs117286316
rs11592078
rs11896747
rs4050551
rs181076711
rs8050653
rs28810746
rs115897087
rs6031492
rs138028805
rs35764061
rs73113464
rs2337147
rs77346511
rs67652251
rs115014362
rs77687616
rs34992898
rs247062
rs72867988
All
0.00
0.01
0.02
0.03
0.04
MR leave−one−out sensitivity analysis for
'CD39 on monocyte ' on 'Arginine to citrulline ratio'

Arginine to citrulline ratio

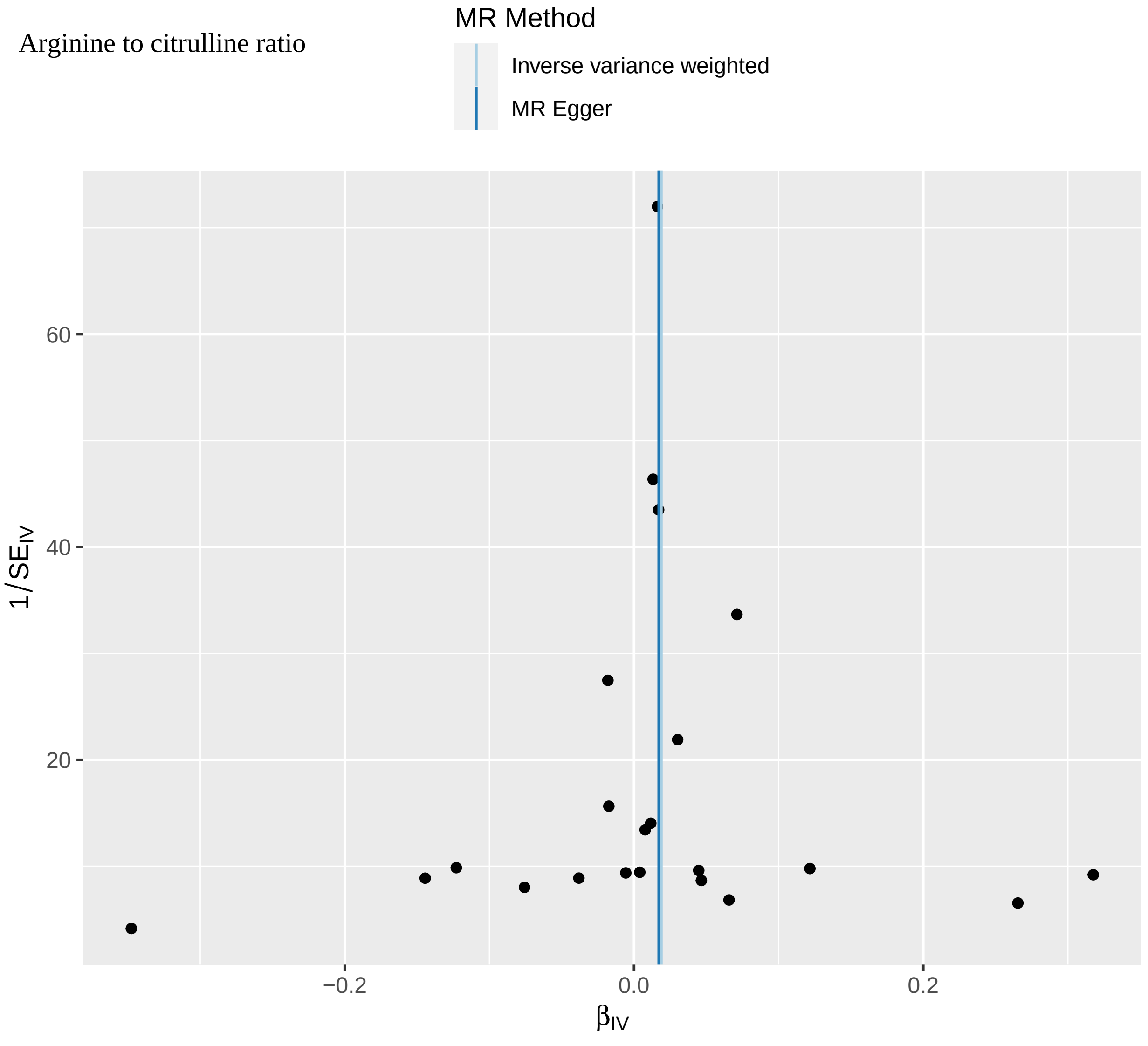

Supplement: Supplementary File 9 — Leave-one-out and Funnel plots to visualize the overall heterogeneity of MR analyses [file DataSheet9.pdf]
